# Identification of Genes Transcriptionally Responsive to the Loss of MLL Fusions in *MLL*-Rearranged Acute Lymphoblastic Leukemia

**DOI:** 10.1371/journal.pone.0120326

**Published:** 2015-03-20

**Authors:** Marieke H. van der Linden, Lidija Seslija, Pauline Schneider, Emma M. C. Driessen, Patricia Garrido Castro, Dominique J. P. M. Stumpel, Eddy van Roon, Jasper de Boer, Owen Williams, Rob Pieters, Ronald W. Stam

**Affiliations:** 1 Department of Pediatric Oncology/Hematology, Erasmus MC—Sophia Children’s Hospital, Rotterdam, The Netherlands; 2 Molecular Haematology and Cancer Biology Unit, University College London Institute of Child Health and Great Ormond Street Hospital for Children, London, United Kingdom; 3 Princess Maxima Center for Pediatric Oncology, Utrecht, The Netherlands; Ghent University, BELGIUM

## Abstract

**Introduction:**

*MLL*-rearranged acute lymphoblastic leukemia (ALL) in infants (<1 year) is characterized by high relapse rates and a dismal prognosis. To facilitate the discovery of novel therapeutic targets, we here searched for genes directly influenced by the repression of various *MLL* fusions.

**Methods:**

For this, we performed gene expression profiling after siRNA-mediated repression of *MLL-AF4*, *MLL-ENL*, and *AF4-MLL* in *MLL*-rearranged ALL cell line models. The obtained results were compared with various already established gene signatures including those consisting of known MLL-AF4 target genes, or those associated with primary *MLL*-rearranged infant ALL samples.

**Results:**

Genes that were down-regulated in response to the repression of *MLL-AF4* and *MLL-ENL* appeared characteristically expressed in primary *MLL*-rearranged infant ALL samples, and often represented known *MLL-AF4* targets genes. Genes that were up-regulated in response to the repression of *MLL-AF4* and *MLL-ENL* often represented genes typically silenced by promoter hypermethylation in *MLL*-rearranged infant ALL. Genes that were affected in response to the repression of *AF4-MLL* showed significant enrichment in gene expression profiles associated with *AF4-MLL* expressing t(4;11)+ infant ALL patient samples.

**Conclusion:**

We conclude that the here identified genes readily responsive to the loss of *MLL* fusion expression potentially represent attractive therapeutic targets and may provide additional insights in *MLL*-rearranged acute leukemias.

## Introduction

A hallmark of acute lymphoblastic leukemia (ALL) in infants (<1 year of age) is a high incidence (∼80%) of chromosomal translocations involving the *Mixed Lineage Leukemia* (*MLL*) gene [1, 2], in which the N-terminal portion of *MLL* fuses to the C-terminal region of one of its many translocation partner genes. [3] The most common *MLL* translocations found among infant ALL patients are t(4;11), t(11;19), and t(9;11), fusing *MLL* to *AF4*, *ENL* and *AF9*, respectively. [2, 4] *MLL*-rearranged infant ALL is associated with an adverse outcome, with event-free survival rates of only ∼30–40%. [2]

*MLL*-rearranged ALL cells display unique gene expression profiles, consisting of overwhelming numbers of differentially transcribed genes [5, 6], which make it difficult to distinguish between the actual “drivers” of the leukemia from the so called “bystanders”. Fortunately, recent advances allowed the identification of genes likely to be activated by MLL fusion proteins via the recruitment of DOT1L. [7–9] Yet, apart from MLL fusion driven activation of gene transcription, inactivation of transcription also plays an important role in *MLL*-rearranged ALL. We and others, recently demonstrated that *MLL*-rearranged infant ALL is characterized by unique patterns of gene promoter DNA hypermethylation, leading to transcriptional silencing of associated genes. [10, 11] To make matters even more complicated, more than half of the t(4;11)-positive ALL patients not only carry the *MLL-AF4* fusion transcript, but also express and translate the reciprocal *AF4-MLL* transcript, which has been proposed to substantially contribute, or even being essential, for leukemia development.[12, 13]

Here, we studied the direct transcriptional consequences of the loss of MLL fusion transcripts in order to identify potential target genes for therapeutic intervention. For this, we performed gene expression profiling in *MLL*-rearranged ALL cell line models in which *MLL-AF4*, *AF4-MLL* or *MLL-ENL* expression was repressed by siRNA-mediated RNA interference. We postulate that genes directly responding to the loss of the MLL fusion represent important therapeutic targets and may provide additional insights into the actions of MLL fusion proteins.

## Methods

Note: More detailed descriptions of all experimental procedures and data analysis methods can be found in the Supplemental Materials (S1 File).

### Cell line models

The B-ALL cell lines RS4;11 and SEMK2 both carry translocation t(4;11) generating the *MLL-AF4* and *AF4-MLL* fusion transcripts. KOPN-8 carries a t(11;19) translocation generating *MLL-ENL* transcripts. RS4;11 was established from the bone marrow of a 32-year-old woman [14], and was purchased from the German Collection of Microorganisms and Cell Cultures (DSMZ). SEMK2 is a subclone of the SEM cell line, which was originally derived from a 5-year-old girl at relapse [15] and was kindly provided by Dr Scott Armstrong (Memorial Sloan Kettering Cancer Center, New York, USA). KOPN-8 was derived from a 3-month-old infant girl with B-cell precursor ALL and was purchased from DSMZ.[16]

### siRNA-mediated RNA interference

Cells were transfected with siRNAs directed against *MLL-AF4* [17], *AF4-MLL* [13], or *MLL-ENL* (sense 5’-CCAAAAGAAAAGUCUGCCCAG-3; antisense 5’-CUGGGCAGACUUUUCUUUUGGUU-3’), using electroporation. Control cells were transfected with siRNAs against *AML1-MTG8* (*AGF1*) [18], a fusion transcript absent in both SEMK2 and RS4;11 cells. For the knock-down of *MLL-AF4* and *MLL-ENL*, cells were harvested after two days. For the *AF4-MLL* knock-down, cells were transfected with siRNAs a second time after two days of culturing, and eventually harvested at day 4. All experiments were performed at least three times.

### RNA extraction

Total RNA was extracted from a minimum of 2x10^6^ cells using TRIzol reagent (Invitrogen, Life Technologies, Breda, The Netherlands) according to manufacturer’s guidelines.

### Gene expression profiling

Gene expression profiling was performed using HU133plus2.0 microarrays (Affymetrix) according to manufacturer’s guidelines. Gene expression profiles for the primary infant ALL patients samples were generated and published previously [19].

## Results

### Transcriptional consequences of MLL fusion knock-down

**Fig 1:**
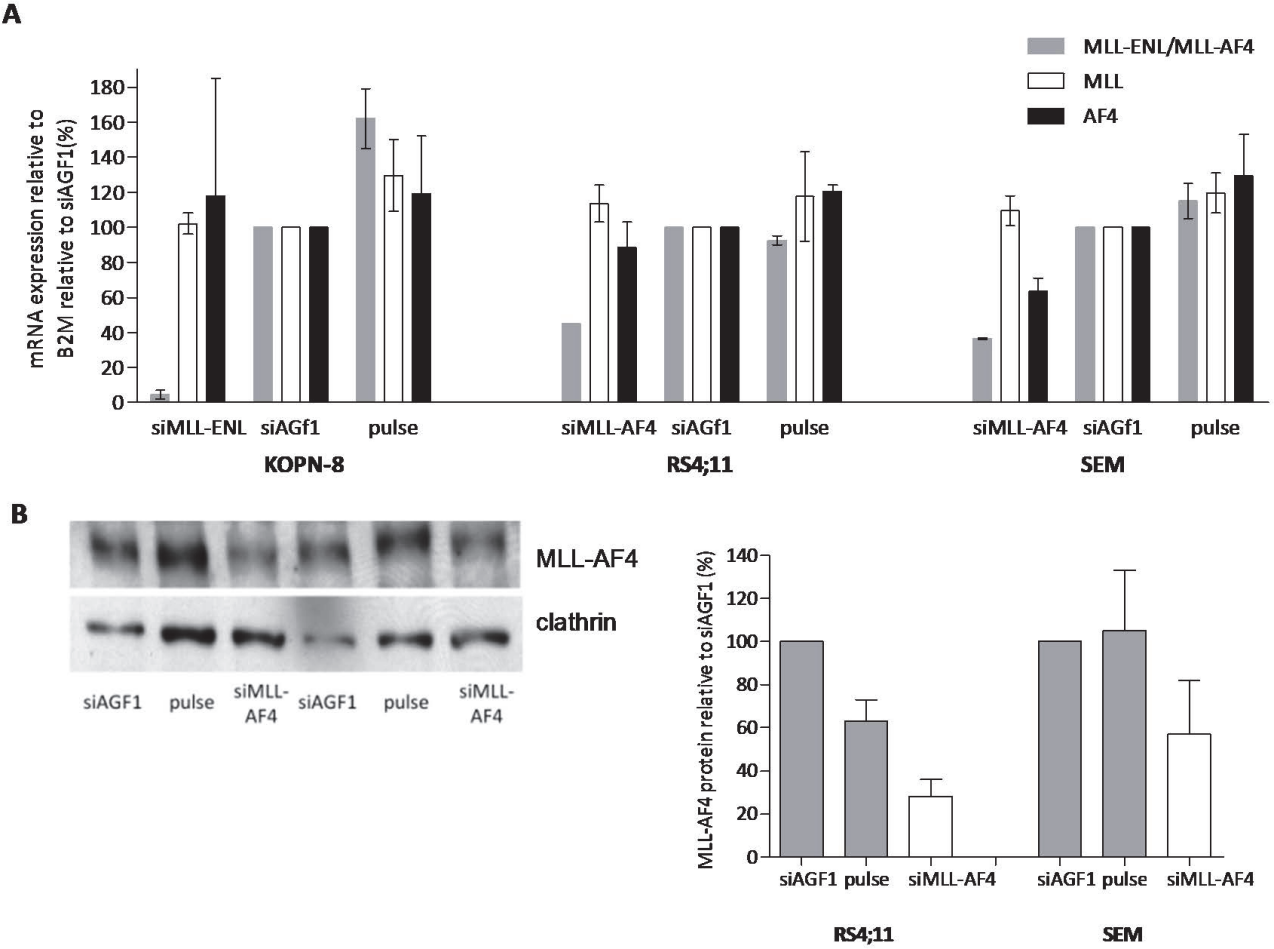
siRNA-mediated knock-down significantly decreases MLL fusion expression levels. (A) mRNA expression levels of *MLL-ENL* (grey), wild-type *MLL* (white), and wild-type *ENL* (black) in KOPN-8 cells, or *MLL-AF4* (grey), wild-type *MLL* (white) and wild-type *AF4* (black) in RS4;11 and SEMK2 cells after transfection with active siRNA directed against the absent target *AML1/MTG8* (si*AGF1*), empty pulse (no siRNAs), and siRNAs directed against *MLL-ENL* and *MLL-AF4* respectively. Shown is the average mRNA expression of two experiments ± standard error of the mean. (B) Protein expression levels of MLL-AF4 in RS4;11 and SEMK2 shown by western blot (left panel). The western blot was probed with antibodies against the N-terminus of MLL to detect MLL-AF4. Clathrin was used as a loading control. The graph (right panel) shows western blot quantification of MLL-AF4 protein expression relative to clathrin with the empty pulse control set at 100%.

Compared to cells transfected with control siRNAs, *MLL-AF4* mRNA expression was reduced to 45% and 37% in the t(4;11)-positive ALL cell lines RS4;11 and SEMK2, respectively, upon transfection with siRNAs directed against *MLL-AF4*. Using siRNAs directed against *MLL-ENL*, the level of *MLL-ENL* expression in KOPN-8 cells was reduced to 5% (Fig. 1A). Western blot analysis demonstrated a reduction of the MLL-AF4 protein expression (relative to control cells) of 28% and 52% in RS4;11 and SEMK2 cells, respectively (Fig. 1B).

**Fig 2:**
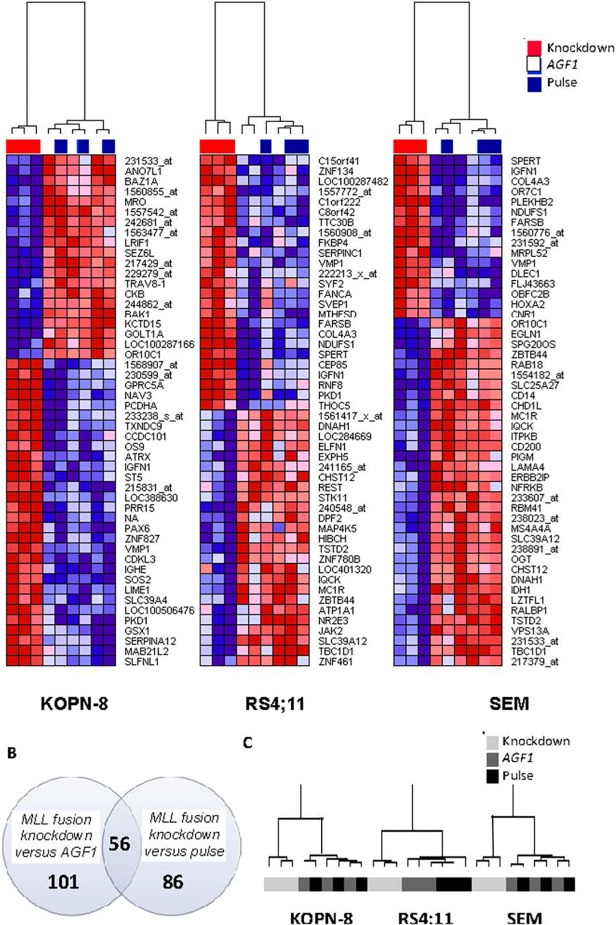
Differential gene expression in response to the repression of MLL-AF4 and MLL-ENL. (A) Heatmap visualization of gene expression profiles (Affymetrix HGU133plus2.0 GeneChips) of the top 50 most differentially expressed genes in response to *MLL-ENL* knock-down in KOPN-8 cells, and *MLL-AF4* knock-down in RS4;11 and SEMK2 cells, as compared to control cells transfected with si*AGF1* or electroporated in the absence of siRNAs (pulse control). The presented data was derived from samples obtained from three independent experiments. Red depicts high expression, blue depicts low expression. (B) Venn diagram showing the number of differentially expressed genes (p<0.001) in KOPN-8, RS4;11, and SEMK2 cells transfected with siRNAs against *MLL-ENL* and *MLL-AF4*, combined (*MLL* fusion knock-down, n = 9) versus control cells transfected with si*AGF1* (*AGF1* control, n = 9), versus control cells electroporated in the absence of siRNAs (pulse control n = 9). All probe sets and gene symbols are listed in S1 and S2 Tables. (C) Hierarchical clustering based on 56 differentially expressed probe sets recurrently affected in both KOPN-8, RS4;11, and SEMK2 cells upon *MLL* fusion knock-down (light grey), in control samples transfected with si*AGF1* (dark grey), and control samples electroporated in the absence of siRNAs (black).

Next, in order to identify genes directly responding to the loss of the MLL fusion, we generated gene expression profiles (HU133plus2.0 GeneChips, Affymetrix) in three independent experiments. Upon repression of *MLL-ENL* in KOPN-8 cells, significant differential expression was observed for 342 probe sets (p<0.001). Reduced expression of *MLL-AF4* resulted in significantly (p<0.001) altered expression of 26 probe sets in RS4;11 cells, and 145 probe sets in SEMK2 cells. Fig. 2A shows heatmaps displaying the most significantly altered probe sets for all three cell lines.

**Fig 3:**
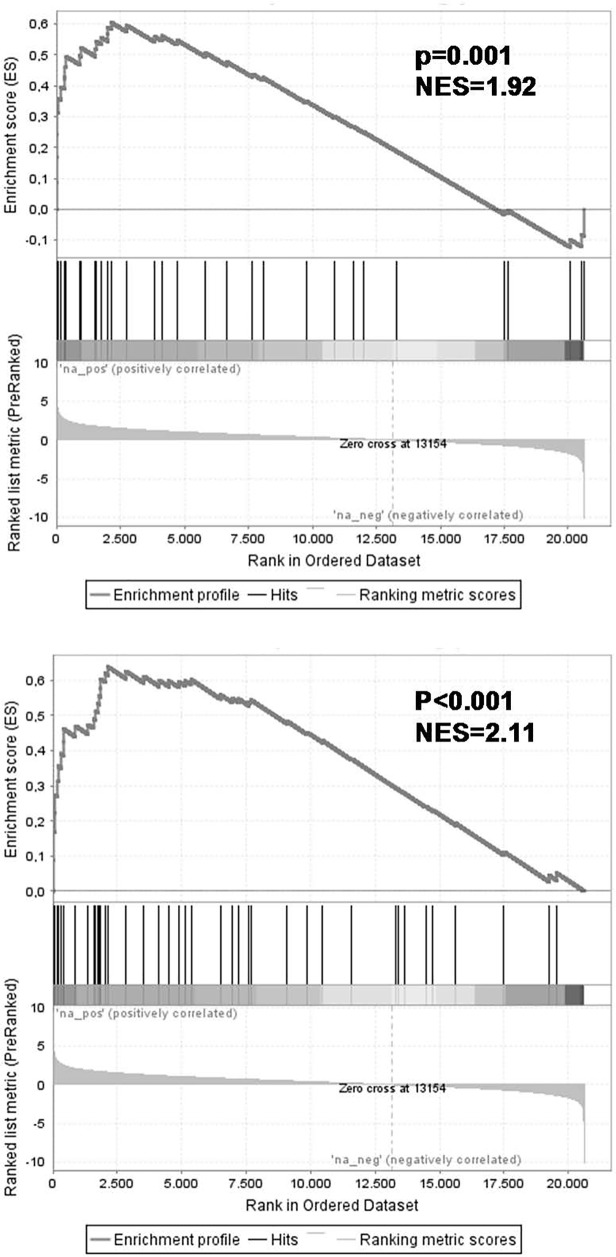
Genes responsive to the repression of MLL-ENL and MLL-AF4 often represent known MLL-AF4 target genes. Gene set enrichment analysis (GSEA) of the 56 differentially expressed probe sets recurrently affected in both KOPN-8, RS4;11, and SEMK2 cells upon *MLL* fusion knock-down in gene lists consisting of MLL-AF4 target genes as published by Guenther et al. (8) (left) and Krivtsov et al. (7) (right). NES = normalized enrichment score.

We searched for a core signature of genes consistently affected in all cell lines by performing a paired analysis of all samples in which the MLL fusion was suppressed (including SEMK2, RS4;11, and KOPN8), compared to all control samples, including cells transduced with control siRNAs directed against *AML1-MTG8* (*AFG1*), as well as control cells electroporated in the absence of siRNAs. Compared to cells transfected with control siRNAs, 101 probe sets appeared to be recurrently affected in all cell lines. Compared to cells only subjected to electroporation in the absence of siRNAs (i.e. pulse control), 86 probe sets were differentially expressed. Merging these analyses, we found 56 overlapping probe sets to be recurrently differentially expressed in all cell lines in which the MLL fusions were suppressed (Fig. 2B). Hierarchical clustering analysis showed that these 56 probe sets effectively distinguished between cells in which the MLL fusion was knocked down and control samples, as separately shown for each cell line (Fig. 2C). As these probe sets consistently responded to the loss of the MLL fusion, we postulate that this gene signature represents genes which are highly dependent on the presence of the MLL fusion, and as such may exert prominent functions in MLL fusion driven transformation. Probe set IDs, HGNC gene symbols, and log-fold changes of the obtained core signature consisting of the 56 probe sets are listed in the supplemental (S1 and **S2** Tables). HGNC gene symbols from these 56 probe sets can also be found next to the heatmap in Fig. 3.

### Relevance of the MLL fusion knock-down signature

To explore the relevance of the obtained gene signature consisting of 56 probe sets responsive to the knock-down of the MLL fusion, we compared our signature to that of earlier published gene sets associated with *MLL*-rearranged ALL. The first signature, published by Guenther *et al* [8], contains 42 genes occupied by the MLL-AF4 fusion protein. The second gene set published by Krivtsov *et al* [7] consists of genes associated with MLL fusion mediated H3K79 dimethylation. Gene set enrichment analysis (GSEA) showed significant enrichment of these genes in our MLL fusion knock-down gene signature (normalized enrichment scores (NES) of 1.92 and 2.11 respectively; leading edge in S3 Table and S4 Table respectively) (Fig. 3). These data indicate that some, but not all, genes transcriptionally activated by the MLL fusion gene itself, rapidly respond to the loss of the MLL fusion.

**Fig 4:**
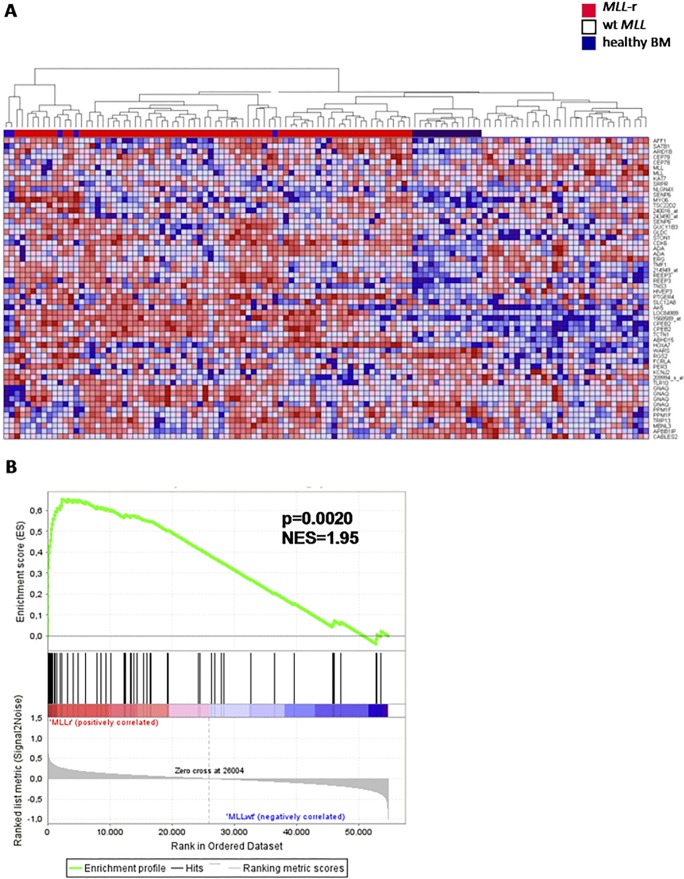
Genes responsive to the repression of MLL-ENL and MLL-AF4 accurately characterize primary *MLL*-rearranged infant ALL samples. (A) Heatmap visualization and hierarchical clustering of primary *MLL*-rearranged infant ALL samples (*MLL*-r, red, n = 71), wild-type *MLL* pediatric ALL samples (both infants (n = 20) and children >1 year of age (n = 16)) (wt *MLL*, blue, n = 36), and whole bone marrow samples derived from healthy children (healthy BM, dark blue, n = 13) based on the 56 differentially expressed probe sets recurrently affected in both KOPN-8, RS4;11, and SEMK2 cells upon *MLL* fusion knock-down. Up-regulated genes are depicted in red, down-regulated genes are depicted in blue. (B) Gene set enrichment analysis (GSEA) of the 57 probe sets that are significantly lower expressed upon knock-down of the *MLL* fusion in gene expression profiles of *MLL*-rearranged patients. Probe sets and HGNC Gene Symbols of the gene set are listed in S5 Table.

Moreover, using our core signature consisting of 56 probe sets associated with knock-down of the MLL fusion, we performed hierarchical clustering on gene expression profiling data generated on a large cohort of primary *MLL*-rearranged infant ALL (n = 71), wild-type *MLL* pediatric precursor B-ALL (n = 16), and wild-type *MLL* infant ALL (n = 20) samples, as well as healthy bone marrow samples (n = 13) as non-leukemic controls. Based on these 56 probe sets, *MLL*-rearranged ALL samples could almost be flawlessly separated from ALL samples with wild-type *MLL* genes (Fig. 4A). These data imply that our MLL fusion knock-down signature represents genes highly characteristic for *MLL*-rearranged ALL. Similarly, gene set enrichment analysis (GSEA) showed strong enrichment in the *MLL*-rearranged patients of the 57 probe sets that are significantly lower expressed after MLL fusion knock-down (NES = 1.95, p = 0.002) (Fig. 4B, dataset in S5 Table; leading edge in S6 Table).

Pathway analysis using the database for annotation, visualization, and integrated discovery (DAVID) demonstrated a significant number of genes transcriptionally responsive to the loss of MLL fusion expression to be involved in the KEGG focal adhesion pathway (hsa04510, p<0.0001) and the KEGG small cell lung cancer pathway (hsa05222, p = 0.008). Next, we used Ingenuity pathway analysis (IPA) to explore possible upstream regulators of the genes down-regulated upon the loss of MLL fusion expression. This revealed 30 potential regulators including 5 genes (i.e. *HOXA7*, *LIN28B*, *UPF1*, *EZH2*, and *MBD1*), and 25 miRNAs (see S7 Table). Interestingly, the majority (i.e. 17 out of the 25) of the miRNAs that potentially regulate the genes down-regulated upon *MLL* fusion knock-down, are predicted to target either *MLL* (i.e. *KMT2A*) or *AF4* (i.e. *AFF1*), or both. Hence, the genes observed to be transcriptionally responsive to the loss of *MLL* fusions, likely represent genes controlled by the MLL fusion itself.

### Transcriptional consequences of AF4-MLL knock-down

**Fig 5:**
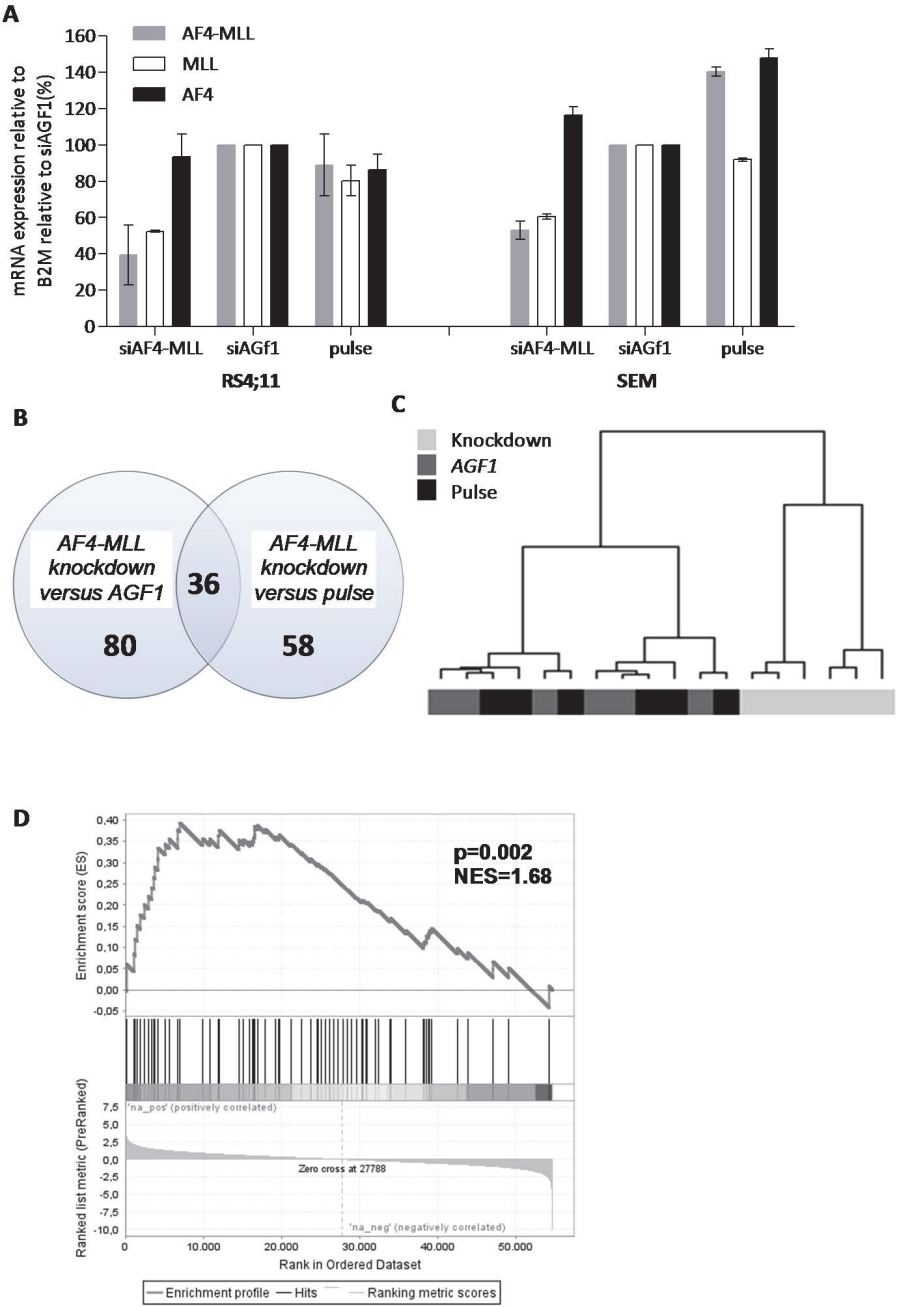
Differential gene expression in response to the repression of *AF4-MLL*. (A) mRNA expression levels of *AF4-MLL* (grey), wild-type *MLL* (white), and wild-type *AF4* (black) in RS4;11 and SEMK2 cells after transfection with siRNAs directed against *AF4-MLL*, siRNAs against the leukemic fusion gene *AML1/MTG8* (si*AGF1*), or cells electroporated in the absence of siRNAs (pulse control). The average mRNA expression relative to the siAGF1 controls of two independent experiments ± standard error of the mean is shown. (B) Venn diagram showing the number of differentially expressed genes (p<0.001) in RS4;11 and SEMK2 cells in which *AF4-MLL* was repressed (*AF4-MLL* knock-down, n = 6), versus cells transfected with control siRNA (si*AGF1* control, n = 6), and cells electroporated in the absence of siRNAs (pulse control, n = 6). (C) Hierarchical clustering based on 36 overlapping differentially expressed probe sets responsive to *AF4-MLL* repression in RS4;11 and SEMK2 (light grey), control samples transfected with si*AGF1* (dark grey), and pulse control samples (black). (D) Gene set enrichment analysis (GSEA) of *AF4-MLL* associated transcription factors (Gaussmann *et al* [20]) in *AF4-MLL* knock-down (‘na_neg’) versus control samples (‘na_pos’). Probe sets and HGNC Gene Symbols are listed in S8 and S9 Tables.

Using siRNAs directed against *AF4-MLL* previously reported by Kumar *et al*. [13], we managed to reduce expression of *AF4-MLL* mRNA to ∼40% in RS4;11 and ∼53% in SEMK2 cells, as compared with cells transfected with control siRNAs (Fig. 5A). In these experiments, the expression of wild-type *AF4* was not affected. However, despite numerous attempts, we were not able to prevent reduction of wild-type *MLL* expression to comparable levels of that of the *AF4-MLL* transcript (Fig. 5A). Paired differential gene expression analysis between samples with a knock-down of *AF4-MLL* (n = 6) and samples transfected with control siRNAs (n = 6) revealed 80 differentially expressed probe sets (p<0.001). The same analysis comparing *AF4-MLL* knock-down samples with control cells electroporated in the absence of siRNAs (i.e. pulse control) (n = 6), revealed 58 differentially expressed probe sets. A total of 36 overlapping probe sets (corresponding to 22 genes) were differentially expressed in both comparisons (Fig. 5B). Probe set IDs, HGNC gene symbols, log-fold changes and p-values are listed in S8 Table and S9 Table. Based on these 36 probe sets, hierarchical clustering could effectively separate *AF4-MLL* knock-down samples from control samples (Fig. 5C).

To validate the *AF4-MLL* knock-down signature, we compared our signature to a gene set published by Gaussmann *et al* consisting of *AF4-MLL* fusion target genes, [20] GSEA showed a significant enrichment of these *AF4-MLL* target genes in our *AF4-MLL* gene signature (NES = 1.68, p = 0.002) (Fig. 5D).

**Fig 6:**
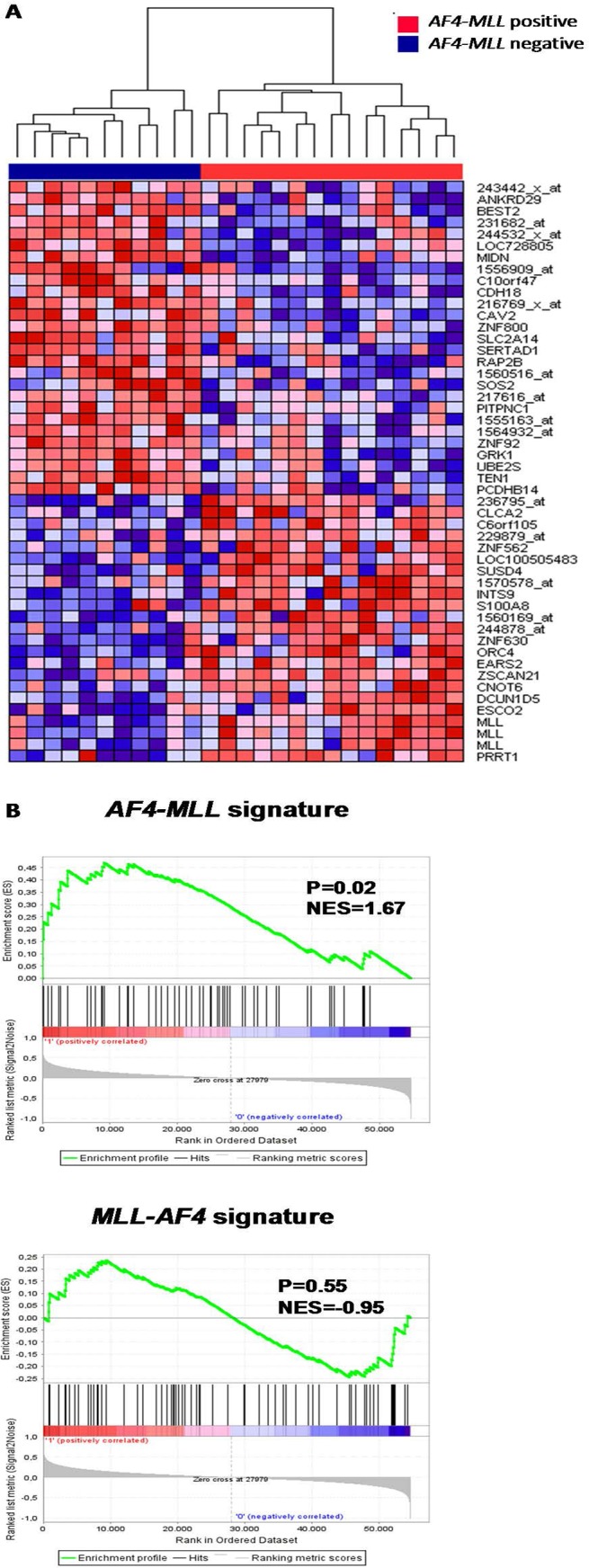
Genes responsive to *AF4-MLL* repression characterize *AF4-MLL* expressing (4;11)^+^ infant ALL patients. (A) Heatmap visualization and hierarchical clustering of primary t(4;11)+ infant ALL samples exhibiting *AF4-MLL* expression (n = 15, red), or lacking *AF4-MLL* expression (n = 11, blue), based on the top 50 most differentially expressed genes between both patient groups. Up-regulated genes are depicted in red, down-regulated genes are depicted in blue. (B) Gene set enrichment analysis (GSEA) of AF4-MLL (upper panel) and MLL-AF4 (lower panel) target genes in *AF4-MLL* positive patients (‘1’) versus *AF4-MLL* negative t(4;11) patients (‘0’). Probe sets and HGNC Gene Symbols are listed in S10 and S11 Tables.

Using our previously published gene expression profiling data, we compared t(4;11)-positive infant ALL samples which do and do not express the reciprocal *AF4-MLL* fusion product, as determined by PCR analysis. This comparison revealed 403 probe sets differentially expressed between both patient groups (p = 0.01). Fig. 6A shows a heatmap of the top 50 most significant differenitially expressed probe sets. Based on these 50 probe sets, principal component analysis (PCA) showed a clear separation of both patient groups (Fig. 6B). Furthermore, we observed that the gene expression patterns of patients expressing the *AF4-MLL* fusion transcript were enriched for genes that were down-regulated after knock-down of the *AF4-MLL* in the cell line models RS4;11 and SEMK2 (GSEA; NES = 1.67, p = 0.02; leading edge in S12 Table) (Fig. 6B, upper panel). As a control we also analyzed enrichment of genes responsive to *AF4-MLL* knock-down in our *MLL-AF4* knock-down signature, and found no significant enrichment (p = 0.55) (Fig. 6B, lower panel).

### Genes up-regulated after MLL fusion knock-down are enriched for hypermethylated promoter regions

**Fig 7:**
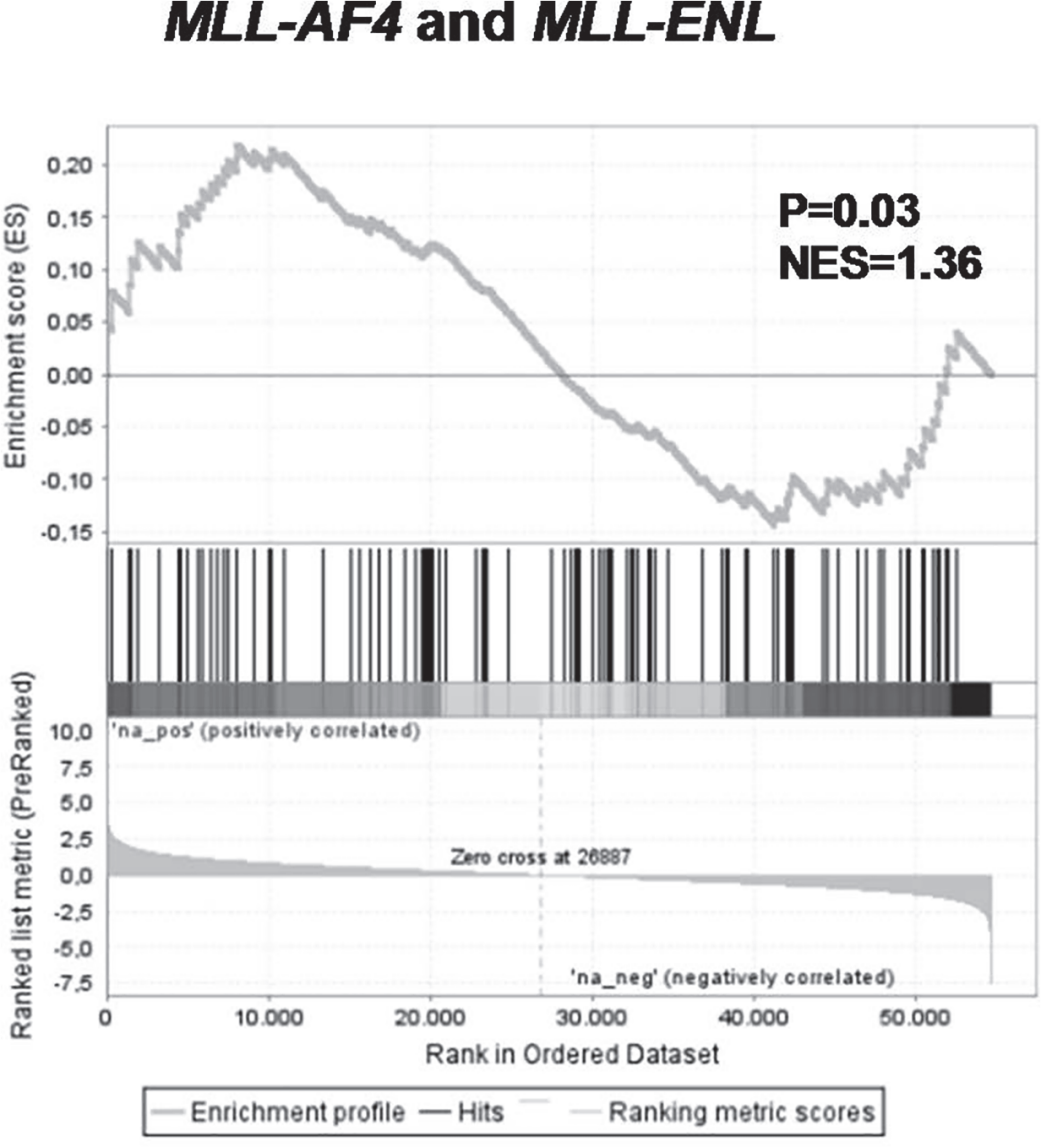
Genes up-regulated in response to *MLL-AF4* and *MLL-ENL* repression include genes normally silenced by promoter methylation in *MLL*-rearranged infant ALL. Gene set enrichment analysis (GSEA) of hypermethylated promoter regions in t(4;11) and t(11;19) patients (Stumpel *et al* [10]) in *MLL* fusion positive (‘na_pos’) versus *MLL* fusion negative (‘na_neg’) samples.

Interestingly, while several known *MLL-AF4* target genes were down-regulated after the loss of the *MLL* fusion, a substantial proportion of genes in our *MLL* fusion knock-down signature were up-regulated (Fig. 2). Per definition these genes are not directly regulated by the MLL fusion protein via H3K79 mediated transcription activation. Therefore, we explored an alternative mechanism of transcriptional regulation. We recently demonstrated that *MLL*-rearranged infant ALL is characterized by severe aberrant DNA hypermethylation, leading to transcriptional silencing of numerous genes. [10] Using 165 probe sets associated with gene promoter methylation in the majority of *MLL*-rearranged infant ALL patients, we applied GSEA on our *MLL* fusion knock-down signatures. GSEA demonstrated significant enrichment of these genes among the genes up-regulated after knock-down of *MLL-AF4* and *MLL-ENL* (NES = 1.36, p = 0.03; leading edge in S13 Table) (Fig. 7). These data suggest that there is an active interplay between MLL fusion proteins and DNA methylation patterns in *MLL*-rearranged ALL cells.

## Discussion

The here identified genes which are transcriptionally responsive to the repression of MLL-AF4 and MLL-ENL represent a rich source of potential therapeutic targets for *MLL*-rearranged acute leukemia. Apart from gene signatures associated with the loss of MLL-AF4 and MLL-ENL, we also identified genes responsive to the repression of AF4-MLL. As it has been suggested that the *AF4-MLL* oncogene could be indispensable for the initiation of t(4;11)^+^ leukemias, our gene signatures associated with the presence and loss of *AF4-MLL* may well provide novel insights into the biology of this leukemia. Apart from genes down-regulated upon the loss of *MLL* fusions, we also identified a substantial number of genes which were up-regulated in response to *MLL* fusion repression. Although MLL fusion proteins activate a variety of target genes (represented in the datasets of Guenther [7, 8] and Krivtsov [7, 8]) by the recruitment of DOT1L and subsequent methylation of H3K79 [7, 8], these data suggest that the MLL fusion itself and/or its activated target genes also actively repress and activate other genes via alternative mechanisms. For instance, we found significant enrichment of the genes activated after knock-down of the *MLL* fusions in our previously published gene signatures associated with promoter hypermethylation in t(4;11)^+^ and t(11;19)^+^ infant ALL samples. [10] This underscores the importance of the role of DNA methylation in *MLL*-rearranged infant ALL, as it appears, to some extent, to be influenced by the presence of the MLL fusion.

Yet, the data presented here should be interpreted with caution as we were not able achieve complete repression of the *MLL* fusions, which may have affected the results. On the other hand, a full knock-down of the *MLL* fusion may not have provided better data per se, as that may have generated more non-specific effects due to enhanced apoptosis induced by the loss of MLL-AF4. [13, 17] Other points of concern may be the slight down-regulation of wild-type *AF4* in SEMK2 cells transfected with siRNAs against *MLL-AF4*, as well as the fact that siRNAs directed against AF4-MLL also affected wild-type *MLL* expression. A possible explanation for this phenomenon is that *MLL* and *AF4* are downstream effectors of the fusion protein. However, we confirmed significant enrichment of differentially regulated genes upon repression of the different MLL fusions in recently published gene signatures consisting of MLL-AF4 target genes (7,8), and a gene signature associated with the loss of AF4-MLL. (17) Moreover, we also demonstrate that our identified genes accurately characterize *MLL*-rearranged ALL patient samples. Furthermore, Ingenuity pathway analysis (IPA) suggested that potential regulators of our gene signature consisting of genes transcriptionally responsive to the knock-down of MLL fusion genes, involve several miRNAs that supposedly target either *MLL* (i.e. KMT2A) and/or *AF4* (i.e. AFF1). This again implies that our gene signatures indeed consist of genes controlled by MLL fusion proteins. Therefore we believe that the obtained data is valid and informative, despite the limited levels of knock-down and the induced suppression of either AF4 or MLL.

Among the here observed genes that are down-regulated after knockdown of the MLL fusions we found several genes that potentially play important roles in leukemia maintenance and/or leukemogenesis. For instance, cyclin-dependent kinase 6 (*CDK6)*, which was identified as one of the *MLL* fusion target genes, as the genomic region encompassing *CDK6* revealed enhanced occupancy of both MLL-AF4 and H3K79 dimethylation.[8] We recently reported data showing the important role of CDK6 in the proliferation of *MLL*-rearranged ALL cells, demonstrating experimentally that inhibition of CDK6 readily induces impairment of leukemic cell proliferation. [21] Other genes in our core signature of genes transcriptionally responsive to the loss of MLL fusion expression are potentially important in leukemogenesis. For example, high expression of the Ets family transcription factor ERG, which is down-regulated after MLL fusion knock-down, is associated with a poor clinical outcome in both acute myeloid leukemia (AML) and T-cell acute lymphoblastic leukemia (T-ALL). [22, 23] Moreover, enforced ERG expression induces both T-ALL and AML in murine models [24, 25], suggesting that ERG contributes to leukemia development. As the inhibition of ERG could possibly benefit the survival of *MLL*-rearranged ALL patients as well as patients suffering from AML and T-ALL, this gene represents an interesting candidate target gene for therapeutic intervention. More genes that are down-regulated after knockdown of the MLL fusions, and that have been associated with oncogenesis include SATB1 [26–28], KAT7 [29–31], ADA [32], PPM1F [33, 34], and HOXA7 [35].

Likewise, potential therapeutic targets can also be found among genes that are up-regulated upon knock-down of the MLL fusions. Among these genes we found regulator of G-protein signaling protein-2 (*RGS2*), which has been demonstrated to contribute to myeloid differentiation and its repression is considered to be an important event in leukemic transformation of FLT3-ITD^+^ AML. [36] Moreover, RGS2 functions as a tumor suppressor in various human cancers. [37–39] Hypothetically, induction of this protein may suppress *MLL*-rearranged ALL progression.

In conclusion, we strongly believe that the genes identified in the present study represent genes which directly and readily respond to the loss of the MLL-AF4, MLL-ENL, or AF4-MLL, and that these genes potentially include attractive therapeutic targets and provide important insights into the biology underlying *MLL*-rearranged acute leukemias.

## Supporting Information

S1 File Materials and Methods. (DOCX)

S1 Table Differentially expressed genes in response to the repression of MLL-AF4 and MLL-ENL as compared to the si*AGF1* control or the pulse control. (DOCX)

S2 Table Differentially expressed genes in response to the repression of MLL-AF4 and MLL-ENL as compared to the si*AGF1* control and the pulse control combined. (DOCX)

S3 Table Leading edge of GSEA comparing MLL-fusion knockdown samples versus control samples using MLL-AF4 target genes dataset from Guenther *et al*. (DOCX)

S4 Table Leading edge of GSEA comparing MLL-fusion knockdown samples versus control samples using MLL-AF4 target genes from Krivtsov *et al*. (DOCX)

S5 Table Differentially lower expressed genes in response to the repression of MLL-AF4 and MLL-ENL as compared to the si*AGF1* control and the pulse control combined. (DOCX)

S6 Table Leading edge of GSEA comparing *MLL*-rearranged patients versus wild-type *MLL* patients using 57 MLL-AF4 target gene probe sets. (DOCX)

S7 Table Upstream regulators of the differentially lower expressed genes in response to the repression of MLL-AF4 and MLL-ENL. (DOCX)

S8 Table Differentially expressed genes in response to the repression of MLL-AF4 and MLL-ENL as compared to the si*AGF1* control or the pulse control. (DOCX)

S9 Table Differentially expressed genes in response to the repression of MLL-AF4 and MLL-ENL as compared to the si*AGF1* control and the pulse control combined. (DOCX)

S10 Table Down-regulated genes in *AF4-MLL* signature. (DOCX)

S11 Table Down-regulated genes in *MLL-AF4* signature. (DOCX)

S12 Table Leading edge of GSEA comparing AF4-MLL positive patients versus AF4-MLL negative t(4;11) patients using 58 AF4-MLL target gene probe sets. (DOCX)

S13 Table Leading edge of GSEA comparing MLL fusion knockdown versus control samples using dataset from Stumpel *et al*. (DOCX)
