# Supplementary material for: Identification of Genes Transcriptionally Responsive to the Loss of MLL Fusions in MLL-Rearranged Acute Lymphoblastic Leukemia
Source: PLoS One. 2015 Mar 20;10(3):e0120326. doi: 10.1371/journal.pone.0120326 (PMC4368425; doi:10.1371/journal.pone.0120326)
Supplement: S1 File — (DOCX) [file pone.0120326.s001.docx]

Methods and Materials

Cell culturing

Leukemia cell lines were maintained as suspension cultures in RPMI 1640 with glutamax (Invitrogen, Life Technologies) supplemented with 10% (v/v) FCS and 2% penicillin/streptomycin/fungizone (PSF; Invitrogen, Life Technologies) at 37°C in humidified air containing 5% CO_2_.

## Transfection with siRNA

4x10^6^ cells were transfected by electroporation in 4 mm electroporation cuvettes (Bio-Rad Laboratories, Benicia, USA) 400 μL of RPMI medium plus 10% fetal calf serum together with 10 μL of esiRNA (20 μM) directed against *MLL-AF4 (*si*MA6) (*[*1*](#_ENREF_1)*), AML1-MTG8* fusion protein (si*AGF1*) as an active siRNA control which is non-silencing in this cellular context (as described previously ([2](#_ENREF_2))), *AF4-MLL* (as described previously ([3](#_ENREF_3))), or 50 μL siRNA (20 μM) directed against *MLL-ENL*: sense 5’-CCAAAAGAAAAGUCUGCCCAG-3; antisense 5’-CUGGGCAGACUUUUCUUUUGGUU-3’ with 50 μL si*AGF1* (20 μM) as an active non-silencing control . siRNAs were purchased from Eurogentec (Seraing, Belgium). Electroporation was performed with the Gene Pulser MX cell Electroporation System (Bio-Rad Laboratories, Benicia, USA) with a rectangle pulse of 350 V for 10 milliseconds. After incubating for 15 minutes at room temperature, the cells were diluted to 1x10^6^ cells/ml and cultured under standard culture conditions. Cells transfected with siRNAs directed against *MLL-AF4* and *MLL-ENL* and the relative controls were harvested after two days. Cells transfected with siRNAs directed against *AF4-MLL* and the relative controls were repeatedly transfected by electroporation after two days under the same conditions and harvested at day 4. All knock-down experiments were performed at least three times.

## RNA extraction and cDNA synthesis

Total RNA was extracted from a minimum of 2x10^6^ cells using TRIzol reagent (Invitrogen, Life Technologies, Breda, The Netherlands) according to the manufacturer’s guidelines. The quality of the extracted RNA was assessed on 1.5% agarose gels and cDNA was prepared for quantitative real-time PCR analysis as described earlier ([4](#_ENREF_4)).

## PCR analysis

*MLL-AF4, MLL-ENL* and *AF4-MLL* mRNA expression was quantified by real-time PCR analysis using the DyNAmo SYBR Green qPCR kit (Finnzymes, Espoo, Finland) as described before ([5](#_ENREF_5)). Oligonucleotide primers used for PCR amplification were purchased from Eurogentec (Seraing, Belgium). Primer sequences were as follows: *MLL-AF4* forward (*MLL* exon 8): 5’- CCCCGCCCAAGTATC-3’, reverse (*AF4* exon 5): 5’-GGCGGCCATGAATG-3’; *MLL-ENL* forward (*MLL* exon 8): 5’-CCCCGCCCAAGTATC-3’, reverse (*ENL* exon 7): 5’-GCTCGAAGTCTGAGTCTGA-3’; *AF4-MLL* forward (*AF4* exon 3): 5’-CAGGCCCCTAGTGAATC-3’, reverse (*MLL* exon 12: 5’-TTTCGGCACTTATTACACTC-3’; *MLL* forward (exon 9): 5’-GCAGGCACTTTGAACATC-3’, reverse (exon 11): 5’-AAGGGCTCACAACAGACTT-3’; *AF4* forward (exon 3): 5’-AATCCCCTGAACTGAAAC-3’, reverse (exon 6): 5’-TTTGGGTTACAGAACTGACA-3’; *ENL* forward (exon 6): 5’-CGGCCAAGGACAAGA-3’, reverse (exon 7): 5’-ATGGCTCGAAGTCTGAGT-3’. *B2M* was used as a reference gene: forward: 5’-GGAGCATTCAGACTTGTCTT-3’, reverse: 5’-ATGCGGCATCTTCAAA-3’. t(4;11)+ infant ALL patient samples were screened for the presence of *AF4-MLL* expression using PCR analysis with the following primer sequences: forward: 5’-CTCCCCTCAAAAAGTGTTGC-3’ (*AF4* exon 3), reverse: 5’-CTTTGCCTGGAGTTGTGGAT-3’ (*MLL* exon 13).

## Western blot

The MLL–AF4 fusion protein was detected using medium-sized 5% polyacrylamide gels. Proteins are resolved at 60–80 volt for at least 10–12 hours at room temperature. The blotting procedure was performed overnight at 4°C on nitrocellulose membranes. Blots were incubated with mouse monoclonal anti-MLL^N^/HRX (clone N4.4) (Upstate Biotechnology, Temecula, CA, USA #05–764) and anti-clathrin HC (clone TD.1) (Santa Cruz Biotechnology, Middlesex, UK #sc-12734) as a loading control. Visualization of the antibodies was done using standard procedures. Western blot procedure and quantification was performed twice.

## Gene expression data

RNA was synthesized into biotinylated cRNA. Labeled cRNA was then fragmented and hybridized to HU133plus2.0 GeneChips (Affymetrix) according to the manufacturer’s guidelines. Differential gene expression analysis was performed using linear modeling for microarray data (LIMMA) ([6](#_ENREF_6)) and was performed in the statistical environment R using Bioconductor packages. Heatmaps were generated in Genepattern using Pearson correlation for hierarchical clustering. ([7](#_ENREF_7)) The pediatric precursor B-ALL samples were deposited as GSE13351 ([8](#_ENREF_8)) as part of recently published studies in the National Center for Biotechnology Information Gene Expression Omnibus. ([9](#_ENREF_9)) Gene set enrichment analysis (GSEA) was performed using GSEA software ([10](#_ENREF_10)). GSEA on gene sets which resulted from paired analyses were done on pre-ranked lists. Pathway analysis was done using DAVID bioinformatics ([11](#_ENREF_11)) and Ingenuity Pathway Analysis (IPA, QIAGEN Redwood City, www.qiagen.com/ingenuity). Gene sets that are used throughout the manuscript other than previously published gene sets are listed in the Supplemental data.

References

1. Thomas M, Gessner A, Vornlocher HP, Hadwiger P, Greil J, Heidenreich O. Targeting MLL-AF4 with short interfering RNAs inhibits clonogenicity and engraftment of t(4;11)-positive human leukemic cells. Blood. 2005 Nov 15;106(10):3559-66. PubMed PMID: 16046533. Epub 2005/07/28. eng.

2. Heidenreich O, Krauter J, Riehle H, Hadwiger P, John M, Heil G, et al. AML1/MTG8 oncogene suppression by small interfering RNAs supports myeloid differentiation of t(8;21)-positive leukemic cells. Blood. 2003 Apr 15;101(8):3157-63. PubMed PMID: 12480707. Epub 2002/12/14. eng.

3. Kumar AR, Yao Q, Li Q, Sam TA, Kersey JH. t(4;11) leukemias display addiction to MLL-AF4 but not to AF4-MLL. Leuk Res. 2011 Mar;35(3):305-9. PubMed PMID: 20869771. Pubmed Central PMCID: 3011030. Epub 2010/09/28. eng.

4. Stam RW, Schneider P, Hagelstein JA, van der Linden MH, Stumpel DJ, de Menezes RX, et al. Gene expression profiling-based dissection of MLL translocated and MLL germline acute lymphoblastic leukemia in infants. Blood. 2010 Apr 8;115(14):2835-44. PubMed PMID: 20032505. Epub 2009/12/25. eng.

5. Stam RW, den Boer ML, Meijerink JP, Ebus ME, Peters GJ, Noordhuis P, et al. Differential mRNA expression of Ara-C-metabolizing enzymes explains Ara-C sensitivity in MLL gene-rearranged infant acute lymphoblastic leukemia. Blood. 2003 Feb 15;101(4):1270-6. PubMed PMID: 12406912. Epub 2002/10/31. eng.

6. Wettenhall JM, Smyth GK. limmaGUI: a graphical user interface for linear modeling of microarray data. Bioinformatics. 2004 Dec 12;20(18):3705-6. PubMed PMID: 15297296. Epub 2004/08/07. eng.

7. Reich M, Liefeld T, Gould J, Lerner J, Tamayo P, Mesirov JP. GenePattern 2.0. Nat Genet. 2006 May;38(5):500-1. PubMed PMID: 16642009. Epub 2006/04/28. eng.

8. Den Boer ML, van Slegtenhorst M, De Menezes RX, Cheok MH, Buijs-Gladdines JG, Peters ST, et al. A subtype of childhood acute lymphoblastic leukaemia with poor treatment outcome: a genome-wide classification study. The lancet oncology. 2009 Feb;10(2):125-34. PubMed PMID: 19138562. Pubmed Central PMCID: 2707020.

9. Edgar R, Domrachev M, Lash AE. Gene Expression Omnibus: NCBI gene expression and hybridization array data repository. Nucleic Acids Res. 2002 Jan 1;30(1):207-10. PubMed PMID: 11752295. Pubmed Central PMCID: 99122. Epub 2001/12/26. eng.

10. Subramanian A, Tamayo P, Mootha VK, Mukherjee S, Ebert BL, Gillette MA, et al. Gene set enrichment analysis: a knowledge-based approach for interpreting genome-wide expression profiles. Proc Natl Acad Sci U S A. 2005 Oct 25;102(43):15545-50. PubMed PMID: 16199517. Pubmed Central PMCID: 1239896. Epub 2005/10/04. eng.

11. Huang da W, Sherman BT, Lempicki RA. Systematic and integrative analysis of large gene lists using DAVID bioinformatics resources. Nature protocols. 2009;4(1):44-57. PubMed PMID: 19131956.
