# Supplementary material for: Identification of Genes Transcriptionally Responsive to the Loss of MLL Fusions in MLL-Rearranged Acute Lymphoblastic Leukemia
Source: PLoS One. 2015 Mar 20;10(3):e0120326. doi: 10.1371/journal.pone.0120326 (PMC4368425; doi:10.1371/journal.pone.0120326)
Supplement: S1 Table — (DOCX) [file pone.0120326.s002.docx]

Table 1. Differentially expressed genes in response to the repression of MLL-AF4 and MLL-ENL as compared to the si*AGF1* control (n=101) or the pulse control (no siRNAs) (n=86) (Figure 2B)

101 probe sets: 86 probe sets:

*MLL* fusion KD versus *AGF1* control *MLL* fusion KD versus pulse control

| Probe set | HGNC Gene Symbol | logFC | P.Value |  | Probe set | HGNC Gene Symbol | logFC | P.Value |
| --- | --- | --- | --- | --- | --- | --- | --- | --- |
| 225785_at | REEP3 | 1.13 | 8.84E-11 |  | 225785_at | REEP3 | 0.94 | 2,52E-08 |
| 1552665_at | LOC84989 | 0.95 | 8.30E-09 |  | 1552665_at | LOC84989 | 0.94 | 5,30E-08 |
| 202318_s_at | SENP6 | 0.72 | 1.24E-08 |  | 202318_s_at | SENP6 | 0.66 | 7,36E-08 |
| 200918_s_at | SRPR | -0.51 | 2.36E-07 |  | 1568589_at | NA | 0.66 | 7,52E-08 |
| 1568589_at | NA | -0.66 | 2.59E-07 |  | 200918_s_at | SRPR | 0.49 | 8,34E-08 |
| 204897_at | PTGER4 | 0.43 | 4.77E-07 |  | 202319_at | SENP6 | 0.49 | 7,96E-07 |
| 202319_at | SENP6 | 0.54 | 5.91E-07 |  | 203408_s_at | SATB1 | -0.36 | 8,54E-07 |
| 221045_s_at | PER3 | 0.36 | 3.62E-06 |  | 228774_at | CEP78 | -0.35 | 2,48E-06 |
| 203408_s_at | SATB1 | -0.38 | 3.69E-06 |  | 240016_at | NA | 0.26 | 2,54E-06 |
| 226796_at | ABHD15 | -0.52 | 4.03E-06 |  | 212078_s_at | MLL | -0.38 | 2,64E-06 |
| 235479_at | CPEB2 | -0.28 | 6.65E-06 |  | 226796_at | ABHD15 | -0.49 | 2,70E-06 |
| 240016_at | NA | 0.23 | 9.69E-06 |  | 204304_s_at | PROM1 | -0.45 | 4,19E-06 |
| 37384_at | PPM1F | -0.28 | 1.37E-05 |  | 235479_at | CPEB2 | -0.27 | 5,48E-06 |
| 228774_at | CEP78 | -0.36 | 1.41E-05 |  | 1557985_s_at | CEP78 | -0.30 | 9,22E-06 |
| 203753_at | TCF4 | -0.37 | 1.42E-05 |  | 201924_at | AFF1 | -0.42 | 9,71E-06 |
| 219874_at | SLC12A8 | 0.35 | 1.73E-05 |  | 214949_at | NA | -0.35 | 1,02E-05 |
| 224862_at | GNAQ | 0.73 | 2.35E-05 |  | 204033_at | TRIP13 | -0.34 | 1,32E-05 |
| 235016_at | REEP3 | 0.35 | 3.01E-05 |  | 235016_at | REEP3 | 0.38 | 1,33E-05 |
| 213541_s_at | ERG | -0.40 | 3.52E-05 |  | 224862_at | GNAQ | 0.61 | 1,94E-05 |
| 203216_s_at | MYO6 | -0.37 | 4.60E-05 |  | 219874_at | SLC12A8 | 0.30 | 2,01E-05 |
| 1557985_s_at | CEP78 | -0.32 | 4.86E-05 |  | 226939_at | CPEB2 | -0.47 | 2,06E-05 |
| 207143_at | CDK6 | -0.37 | 5.14E-05 |  | 241926_s_at | ERG | -0.40 | 2,26E-05 |
| 203063_at | PPM1F | -0.24 | 5.76E-05 |  | 213541_s_at | ERG | -0.36 | 2,36E-05 |
| 214949_at | NA | -0.36 | 6.30E-05 |  | 225181_at | ARID1B | 0.37 | 4,15E-05 |
| 235372_at | FCRLA | 0.33 | 6.49E-05 |  | 204897_at | PTGER4 | 0.36 | 5,43E-05 |
| 223750_s_at | TLR10 | 0.24 | 6.68E-05 |  | 238767_at | NA | -0.24 | 7,14E-05 |
| 201924_at | AFF1 | -0.37 | 7.12E-05 |  | 216705_s_at | ADA | -0.40 | 7,27E-05 |
| 208934_s_at | LGALS8 | 0.25 | 7.47E-05 |  | 210571_s_at | CMAHP | 0.28 | 8,87E-05 |
| 1564776_at | NA | -0.30 | 7.69E-05 |  | 219563_at | LINC00341 | -0.23 | 9,46E-05 |
| 204639_at | ADA | -0.42 | 1.04E-04 |  | 235753_at | HOXA7 | -0.38 | 0,000105 |
| 213413_at | STON1 | 0.24 | 1.07E-04 |  | 37384_at | PPM1F | -0.25 | 0,00012 |
| 201889_at | FAM3C | 0.34 | 1.16E-04 |  | 224861_at | GNAQ | 0.52 | 0,000129 |
| 226939_at | CPEB2 | -0.46 | 1.19E-04 |  | 1561707_at | LOC150185 | 0.24 | 0,000143 |
| 200049_at | KAT7 | -0.31 | 1.24E-04 |  | 1567224_at | HMGA2 | -0.24 | 0,000145 |
| 202615_at | GNAQ | 0.57 | 1.32E-04 |  | 207819_s_at | ABCB4 | 0.20 | 0,000159 |
| 207966_s_at | GLG1 | 0.28 | 1.34E-04 |  | 230925_at | APBB1IP | -0.35 | 0,000165 |
| 225181_at | ARID1B | 0.44 | 1.35E-04 |  | 207143_at | CDK6 | -0.35 | 0,000179 |
| 235964_x_at | SAMHD1 | 0.43 | 1.50E-04 |  | 203216_s_at | MYO6 | -0.33 | 0,000181 |
| 224861_at | GNAQ | 0.57 | 1.59E-04 |  | 233931_at | NA | -0.30 | 0,00019 |
| 226004_at | CABLES2 | -0.27 | 1.76E-04 |  | 212079_s_at | MLL | -0.32 | 0,000193 |
| 203817_at | GUCY1B3 | -0.60 | 1.76E-04 |  | 204094_s_at | TSC22D2 | -0.30 | 0,000205 |
| 230925_at | APBB1IP | -0.37 | 1.77E-04 |  | 226004_at | CABLES2 | -0.25 | 0,000231 |
| 201859_at | SRGN | 0.40 | 1.82E-04 |  | 204639_at | ADA | -0.36 | 0,000232 |
| 204304_s_at | PROM1 | -0.43 | 1.83E-04 |  | 224863_at | GNAQ | 0.43 | 0,00024 |
| 225406_at | TWSG1 | 0.30 | 1.91E-04 |  | 206847_s_at | HOXA7 | -0.41 | 0,000243 |
| 214948_s_at | TMF1 | -0.35 | 2.31E-04 |  | 235122_at | HIVEP3 | 0.32 | 0,000248 |
| 204033_at | TRIP13 | -0.30 | 2.42E-04 |  | 218584_at | TCTN1 | 0.34 | 0,000276 |
| 201925_s_at | CD55 | 0.27 | 2.49E-04 |  | 217853_at | TNS3 | 0.42 | 0,000302 |
| 243490_at | NA | -0.42 | 2.62E-04 |  | 214948_s_at | TMF1 | -0.33 | 0,000308 |
| 235529_x_at | SAMHD1 | 0.39 | 2.67E-04 |  | 235919_at | NA | -0.37 | 0,00031 |
| 210480_s_at | MYO6 | -0.33 | 2.70E-04 |  | 202615_at | GNAQ | 0.48 | 0,00032 |
| 211555_s_at | GUCY1B3 | -0.49 | 2.76E-04 |  | 204621_s_at | NR4A2 | 0.16 | 0,000339 |
| AFFX-M27830_M_at | NA | -0.31 | 2.80E-04 |  | 200629_at | WARS | 0.31 | 0,000378 |
| 225355_at | NEURL1B | -0.25 | 2.91E-04 |  | 219497_s_at | BCL11A | 0.22 | 0,000417 |
| 230281_at | C16orf46 | 0.22 | 3.04E-04 |  | 210432_s_at | SCN3A | 0.36 | 0,00045 |
| 216705_s_at | ADA | -0.41 | 3.15E-04 |  | 232544_at | NA | -0.24 | 0,000451 |
| 218584_at | TCTN1 | 0.38 | 3.17E-04 |  | 1553145_at | FLJ39653 | -0.32 | 0,000452 |
| 221933_at | NLGN4X | 0.27 | 3.19E-04 |  | 224906_at | ANO6 | -0.21 | 0,000456 |
| 235753_at | HOXA7 | -0.36 | 3.20E-04 |  | 213413_at | STON1 | 0.24 | 0,000457 |
| 200629_at | WARS | 0.30 | 3.37E-04 |  | 200049_at | KAT7 | -0.26 | 0,000473 |
| 204836_at | GLDC | 0.28 | 3.49E-04 |  | 220459_at | MCM3AP-AS1 | -0.26 | 0,000491 |
| 202723_s_at | FOXO1 | -0.31 | 3.69E-04 |  | 223750_s_at | TLR10 | 0.23 | 0,000527 |
| 202656_s_at | SERTAD2 | -0.27 | 4.00E-04 |  | 205488_at | GZMA | 0.33 | 0,000529 |
| 224882_at | ACSS1 | -0.28 | 4.01E-04 |  | 211965_at | ZFP36L1 | 0.37 | 0,000553 |
| 203860_at | PCCA | 0.20 | 4.27E-04 |  | 229838_at | NUCB2 | 0.22 | 0,000574 |
| 202388_at | RGS2 | 0.44 | 4.37E-04 |  | 225639_at | SKAP2 | -0.34 | 0,000577 |
| 212080_at | MLL | -0.20 | 4.40E-04 |  | 229498_at | MBNL3 | -0.32 | 0,000592 |
| 209994_s_at | NA | 0.19 | 4.50E-04 |  | 225283_at | ARRDC4 | 0.17 | 0,000613 |
| 243001_at | RBFA | -0.34 | 4.60E-04 |  | 212045_at | GLG1 | 0.20 | 0,000623 |
| 213734_at | NA | 0.26 | 4.81E-04 |  | 202388_at | RGS2 | 0.37 | 0,000624 |
| 224863_at | GNAQ | 0.42 | 5.02E-04 |  | 203817_at | GUCY1B3 | -0.54 | 0,000626 |
| 213704_at | RABGGTB | 0.23 | 5.19E-04 |  | 209994_s_at | NA | 0.19 | 0,000668 |
| 235400_at | FCRLA | 0.30 | 5.38E-04 |  | 219498_s_at | BCL11A | -0.21 | 0,00067 |
| 235401_s_at | FCRLA | 0.32 | 5.75E-04 |  | 211962_s_at | ZFP36L1 | 0.38 | 0,000685 |
| 230415_at | NA | -0.21 | 5.82E-04 |  | 206765_at | KCNJ2 | 0.50 | 0,000729 |
| 243879_at | NA | -0.25 | 5.90E-04 |  | 211991_s_at | HLA-DPA1 | -0.47 | 0,000791 |
| 224567_x_at | MALAT1 | 0.43 | 5.94E-04 |  | 204836_at | GLDC | 0.21 | 0,000796 |
| 206765_at | KCNJ2 | 0.47 | 5.95E-04 |  | 203063_at | PPM1F | -0.22 | 0,000801 |
| 235122_at | HIVEP3 | 0.35 | 5.97E-04 |  | 222862_s_at | AK5 | 0.24 | 0,000817 |
| 222862_s_at | AK5 | 0.23 | 6.24E-04 |  | 243490_at | NA | -0.36 | 0,000817 |
| 228496_s_at | CRIM1 | 0.27 | 6.28E-04 |  | 228886_at | LRRC27 | 0.16 | 0,000825 |
| 205006_s_at | NMT2 | 0.18 | 6.71E-04 |  | 235372_at | FCRLA | 0.27 | 0,000827 |
| 236443_at | NA | -0.20 | 6.90E-04 |  | 235292_at | FLJ32255 | 0.25 | 0,000923 |
| 240236_at | STXBP5L | -0.22 | 7.00E-04 |  | 221933_at | NLGN4X | 0.27 | 0,000954 |
| 231812_x_at | PHAX | 0.19 | 7.18E-04 |  | 221045_s_at | PER3 | 0.25 | 0,000962 |
| 243769_at | NA | 0.35 | 7.31E-04 |  |  |  |  |  |
| 212078_s_at | MLL | -0.29 | 7.45E-04 |  |  |  |  |  |
| 228377_at | KLHL14 | 0.27 | 7.48E-04 |  |  |  |  |  |
| 232096_x_at | FOXP1-IT1 | -0.23 | 7.78E-04 |  |  |  |  |  |
| 224993_at | MLLT1 | -0.22 | 7.85E-04 |  |  |  |  |  |
| 209447_at | SYNE1 | 0.26 | 7.88E-04 |  |  |  |  |  |
| 229594_at | SPTY2D1 | 0.19 | 8.09E-04 |  |  |  |  |  |
| 224793_s_at | TGFBR1 | 0.34 | 8.53E-04 |  |  |  |  |  |
| 237173_at | NA | -0.18 | 8.60E-04 |  |  |  |  |  |
| 224699_s_at | ESYT2 | -0.22 | 8.91E-04 |  |  |  |  |  |
| 234723_x_at | NA | 0.22 | 8.92E-04 |  |  |  |  |  |
| 217853_at | TNS3 | 0.38 | 9.05E-04 |  |  |  |  |  |
| 212079_s_at | MLL | -0.31 | 9.44E-04 |  |  |  |  |  |
| 242911_at | MED13L | -0.21 | 9.47E-04 |  |  |  |  |  |
| 226301_at | C6orf192 | 0.23 | 9.56E-04 |  |  |  |  |  |
| 228771_at | ADRBK2 | 0.21 | 9.71E-04 |  |  |  |  |  |
