# Supplementary material for: Identification of Genes Transcriptionally Responsive to the Loss of MLL Fusions in MLL-Rearranged Acute Lymphoblastic Leukemia
Source: PLoS One. 2015 Mar 20;10(3):e0120326. doi: 10.1371/journal.pone.0120326 (PMC4368425; doi:10.1371/journal.pone.0120326)
Supplement: S2 Table — (DOCX) [file pone.0120326.s003.docx]

**Table 2. Differentially expressed genes in response to the repression of MLL-AF4 and MLL-ENL as compared to the si*AGF1* control and the pulse control (no siRNAs) combined (n=56) (Figure 2B)**

| Probe set | HGNC Gene Symbol |
| --- | --- |
| 1552665_at | LOC84989 |
| 1557985_s_at | CEP78 |
| 1568589_at | NA |
| 200049_at | KAT7 |
| 200629_at | WARS |
| 200918_s_at | SRPR |
| 201924_at | AFF1 |
| 202318_s_at | SENP6 |
| 202319_at | SENP6 |
| 202388_at | RGS2 |
| 202615_at | GNAQ |
| 203063_at | PPM1F |
| 203216_s_at | MYO6 |
| 203408_s_at | SATB1 |
| 203817_at | GUCY1B3 |
| 204033_at | TRIP13 |
| 204094_s_at | TSC22D2 |
| 204639_at | ADA |
| 204836_at | GLDC |
| 204897_at | PTGER4 |
| 206765_at | KCNJ2 |
| 207143_at | CDK6 |
| 209994_s_at | NA |
| 212078_s_at | MLL |
| 212079_s_at | MLL |
| 213413_at | STON1 |
| 213541_s_at | ERG |
| 214948_s_at | TMF1 |
| 214949_at | NA |
| 216705_s_at | ADA |
| 217853_at | TNS3 |
| 218584_at | TCTN1 |
| 219874_at | SLC12A8 |
| 221045_s_at | PER3 |
| 221933_at | NLGN4X |
| 222862_s_at | AK5 |
| 223750_s_at | TLR10 |
| 224861_at | GNAQ |
| 224862_at | GNAQ |
| 224863_at | GNAQ |
| 225181_at | ARID1B |
| 225785_at | REEP3 |
| 226004_at | CABLES2 |
| 226796_at | ABHD15 |
| 226939_at | CPEB2 |
| 228774_at | CEP78 |
| 229498_at | MBNL3 |
| 230925_at | APBB1IP |
| 235016_at | REEP3 |
| 235122_at | HIVEP3 |
| 235372_at | FCRLA |
| 235479_at | CPEB2 |
| 235753_at | HOXA7 |
| 240016_at | NA |
| 243490_at | NA |
| 37384_at | PPM1F |
