# Supplementary material for: Identification of Genes Transcriptionally Responsive to the Loss of MLL Fusions in MLL-Rearranged Acute Lymphoblastic Leukemia
Source: PLoS One. 2015 Mar 20;10(3):e0120326. doi: 10.1371/journal.pone.0120326 (PMC4368425; doi:10.1371/journal.pone.0120326)
Supplement: S3 Table — (DOCX) [file pone.0120326.s004.docx]

**Table 3. Leading edge of GSEA comparing MLL-fusion knockdown samples versus control samples using MLL-AF4 target genes from Guenther *et al* (Figure 3, upper panel)**

| HGNC Gene Symbol |
| --- |
| ERG |
| CDK6 |
| PROM1 |
| HOXA7 |
| PPP2R5C |
| ZEB2 |
| GALNT2 |
| UBASH3B |
| HOXA10 |
| TNRC18 |
| SUPT3H |
| JMJD1C |
| CPNE8 |
| ADAM10 |
| BCL7A |
| MEIS1 |
| TWIST1 |
| SENP6 |
