# Supplementary material for: Identification of Genes Transcriptionally Responsive to the Loss of MLL Fusions in MLL-Rearranged Acute Lymphoblastic Leukemia
Source: PLoS One. 2015 Mar 20;10(3):e0120326. doi: 10.1371/journal.pone.0120326 (PMC4368425; doi:10.1371/journal.pone.0120326)
Supplement: S4 Table — (DOCX) [file pone.0120326.s005.docx]

**Table 4. Leading edge of GSEA comparing MLL-fusion knockdown samples versus control samples using MLL-AF4 target genes from Krivtsov *et al* (Figure 3, lower panel)**

| HGNC Gene Symbol |
| --- |
| PROM1 |
| HOXA7 |
| CLEC14A |
| BCL2 |
| SOCS2 |
| RPL32 |
| MAP3K5 |
| HOXA10 |
| HOXA6 |
| CD93 |
| FOSL2 |
| ADCY9 |
| CEBPA |
| ZNRF1 |
| FUT4 |
| MEIS1 |
| C3orf65 |
| HEXB |
| KCNK12 |
| VLDLR |
| MMP17 |
| MRM1 |
| LCN8 |
| GREM1 |
| HTRA3 |
| AKR7A2 |
| SERPINB1 |
| PCDHGC3 |
| RUNX2 |
| CDKN1A |
| NLRP3 |
| CTGF |
| TMEM173 |
