# Supplementary material for: Identification of Genes Transcriptionally Responsive to the Loss of MLL Fusions in MLL-Rearranged Acute Lymphoblastic Leukemia
Source: PLoS One. 2015 Mar 20;10(3):e0120326. doi: 10.1371/journal.pone.0120326 (PMC4368425; doi:10.1371/journal.pone.0120326)
Supplement: S5 Table — (DOCX) [file pone.0120326.s006.docx]

**Table 5. Differentially lower expressed genes in response to the repression of MLL-AF4 and MLL-ENL as compared to the si*AGF1* control and the pulse control (no siRNAs) combined (n=57) (Figure 4B)**

| Probe set | HGNC Gene Symbol |
| --- | --- |
| 1553145_at | FLJ39653 |
| 1557985_s_at | CEP78 |
| 1564776_at | NA |
| 1567224_at | HMGA2 |
| 1568589_at | NA |
| 200049_at | MYST2 |
| 200918_s_at | SRPR |
| 201924_at | AFF1 |
| 202656_s_at | SERTAD2 |
| 203063_at | PPM1F |
| 203216_s_at | MYO6 |
| 203408_s_at | SATB1 |
| 203753_at | TCF4 |
| 203817_at | GUCY1B3 |
| 204033_at | TRIP13 |
| 204082_at | PBX3 |
| 204094_s_at | TSC22D2 |
| 204304_s_at | PROM1 |
| 204639_at | ADA |
| 206847_s_at | HOXA7 |
| 207143_at | CDK6 |
| 210480_s_at | MYO6 |
| 211555_s_at | GUCY1B3 |
| 211991_s_at | HLA-DPA1 |
| 212078_s_at | MLL |
| 212079_s_at | MLL |
| 212080_at | MLL |
| 213541_s_at | ERG |
| 214948_s_at | TMF1 |
| 214949_at | NA |
| 216705_s_at | ADA |
| 219498_s_at | BCL11A |
| 219563_at | C14orf139 |
| 223840_s_at | SPATA9 |
| 224699_s_at | ESYT2 |
| 224882_at | ACSS1 |
| 224906_at | ANO6 |
| 225355_at | NEURL1B |
| 226004_at | CABLES2 |
| 226796_at | ABHD15 |
| 226939_at | CPEB2 |
| 228774_at | CEP78 |
| 229498_at | NA |
| 230415_at | NA |
| 230925_at | APBB1IP |
| 232544_at | NA |
| 233931_at | NA |
| 235479_at | CPEB2 |
| 235753_at | HOXA7 |
| 235919_at | NA |
| 236443_at | NA |
| 238767_at | NA |
| 240236_at | STXBP5L |
| 243001_at | C18orf22 |
| 243490_at | NA |
| 243879_at | NA |
| 37384_at | PPM1F |
