# Supplementary material for: Identification of Genes Transcriptionally Responsive to the Loss of MLL Fusions in MLL-Rearranged Acute Lymphoblastic Leukemia
Source: PLoS One. 2015 Mar 20;10(3):e0120326. doi: 10.1371/journal.pone.0120326 (PMC4368425; doi:10.1371/journal.pone.0120326)
Supplement: S6 Table — (DOCX) [file pone.0120326.s007.docx]

**Table 6. Differentially expressed genes in response to the repression of MLL-AF4 and MLL-ENL as compared to the si*AGF1* control and the pulse control (no siRNAs) combined (n=36) (Figure 5C)**

| Probe set | HGNC Gene Symbol |
| --- | --- |
| 1552726_at | ADAMTS17 |
| 204249_s_at | LMO2 |
| 208724_s_at | RAB1A |
| 209789_at | CORO2B |
| 211924_s_at | PLAUR |
| 212080_at | MLL |
| 212262_at | QKI |
| 212636_at | QKI |
| 212750_at | PPP1R16B |
| 213708_s_at | MLX |
| 214743_at | CUX1 |
| 214866_at | PLAUR |
| 217910_x_at | MLX |
| 219326_s_at | B3GNT2 |
| 222631_at | PI4K2B |
| 222870_s_at | B3GNT2 |
| 222942_s_at | NA |
| 222958_s_at | DEPDC1 |
| 223017_at | TXNDC12 |
| 223171_at | DYM |
| 224967_at | UGCG |
| 225935_at | CUX1 |
| 226297_at | HIPK3 |
| 226689_at | CISD2 |
| 226793_at | LINC00294 |
| 227069_at | CUX1 |
| 228008_at | NA |
| 228094_at | AMICA1 |
| 228486_at | SLC44A1 |
| 232278_s_at | DEPDC1 |
| 233727_at | NA |
| 235545_at | DEPDC1 |
| 236513_at | NA |
| 238041_at | TCF12 |
| 239400_at | NA |
| 41577_at | PPP1R16B |
