# Supplementary material for: Identification of Genes Transcriptionally Responsive to the Loss of MLL Fusions in MLL-Rearranged Acute Lymphoblastic Leukemia
Source: PLoS One. 2015 Mar 20;10(3):e0120326. doi: 10.1371/journal.pone.0120326 (PMC4368425; doi:10.1371/journal.pone.0120326)
Supplement: S7 Table — (DOCX) [file pone.0120326.s008.docx]

**Table 7. Upstream regulators of the differentially lower expressed genes in response to the repression of MLL-AF4 and MLL-ENL.**

| Upstream Regulator | Molecule Type | p-value of overlap | Target molecules in dataset |
| --- | --- | --- | --- |
| miR-92a-3p | mature microrna | 9.39E-08 | AFF1,BCL11A,CDK6,CPEB2,HMGA2,MBNL3,NEURL1B,  SERTAD2,SRPR,STXBP5L,TCF4,TMF1 |
| mir-196 | microrna | 1.97E-06 | ERG,HMGA2,HOXA7 |
| miR-344d-3p | mature microrna | 2.65E-06 | AFF1,BCL11A,CPEB2,ERG,HMGA2,MBNL3,PBX3,SRPR,  TCF4 |
| miR-144-3p | mature microrna | 6.57E-06 | BCL11A,CPEB2,HOXA7,KAT7,MBNL3,NEURL1B,PBX3,  STXBP5L,TCF4,TMF1 |
| miR-153-3p | mature microrna | 1.07E-05 | AFF1,BCL11A,CPEB2,KMT2A,MBNL3,SATB1,SERTAD2,  STXBP5L,TCF4 |
| miR-219a-5p | mature microrna | 1.18E-05 | BCL11A,CPEB2,ERG,HMGA2,SATB1,STXBP5L,TSC22D2 |
| miR-17-5p | mature microrna | 1.48E-05 | AFF1,ANO6,CDK6,HMGA2,KMT2A,MBNL3,NEURL1B,  PBX3,SERTAD2,TCF4,TSC22D2 |
| HOXA7 | transcription regulator | 1.76E-05 | ERG,HOXA7,SATB1 |
| miR-196a-5p | mature microrna | 4.21E-05 | BCL11A,ERG,HMGA2,HOXA7,NEURL1B,PBX3 |
| miR-137-3p | mature microrna | 4.87E-05 | BCL11A,CABLES2,CDK6,ERG,KMT2A,SRPR,STXBP5L,  TCF4,TMF1,TSC22D2 |
| MIRLET7 | group | 5.61E-05 | BCL11A,HMGA2 |
| mir-142 | microrna | 5.61E-05 | HMGA2,PROM1 |
| miR-142-3p | mature microrna | 6.91E-05 | AFF1,CPEB2,ERG,HMGA2,KAT7,PROM1 |
| miR-148a-3p | mature microrna | 7.82E-05 | BCL11A,CDK6,HMGA2,KAT7,KMT2A,NEURL1B,  STXBP5L,TCF4 |
| miR-590-3p | mature microrna | 8.92E-05 | AFF1,CDK6,CPEB2,HMGA2,MBNL3,MYO6,PPM1F,  SATB1,STXBP5L,TMF1 |
| LIN28B | other | 1.34E-04 | BCL11A,PROM1 |
| miR-3922-5p | mature microrna | 1.63E-04 | ABHD15,BCL11A,ERG,ESYT2 |
| miR-154-5p | mature microrna | 1.72E-04 | HMGA2,KAT7,MYO6,STXBP5L |
| miR-186-5p | mature microrna | 1.90E-04 | AFF1,ANO6,CDK6,CPEB2,SATB1,SERTAD2,TMF1,TSC22D2 |
| UPF1 | enzyme | 2.89E-04 | ERG,HMGA2 |
| miR-191-5p | mature microrna | 3.79E-04 | BCL11A,CDK6,SATB1 |
| miR-448-3p | mature microrna | 3.79E-04 | BCL11A,CPEB2,MBNL3,SATB1,SERTAD2,STXBP5L,TCF4 |
| miR-342-3p | mature microrna | 4.49E-04 | AFF1,CDK6,KMT2A,MBNL3,NEURL1B |
| miR-9-5p | mature microrna | 4.87E-04 | AFF1,ANO6,BCL11A,CPEB2,ERG,ESYT2,HMGA2,PPM1F,  TSC22D2 |
| miR-21-5p | mature microrna | 5.42E-04 | BCL11A,CDK6,ERG,MBNL3,SATB1 |
| miR-155-5p | mature microrna | 7.12E-04 | MBNL3,SATB1,SERTAD2,STXBP5L,TCF4,TRIP13 |
| EZH2 | transcription regulator | 8.23E-04 | CABLES2,CDK6,HOXA7,SATB1 |
| miR-200b-3p | mature microrna | 8.47E-04 | AFF1,ANO6,ERG,MBNL3,PBX3,PPM1F,TCF4,TSC22D2 |
| miR-128-3p | mature microrna | 8.53E-04 | AFF1,CABLES2,ERG,KAT7,KMT2A,NEURL1B,SERTAD2,  TCF4 |
| MBD1 | transcription regulator | 9.26E-04 | HOXA7,PROM1 |
