# Supplementary material for: Identification of Genes Transcriptionally Responsive to the Loss of MLL Fusions in MLL-Rearranged Acute Lymphoblastic Leukemia
Source: PLoS One. 2015 Mar 20;10(3):e0120326. doi: 10.1371/journal.pone.0120326 (PMC4368425; doi:10.1371/journal.pone.0120326)
Supplement: S8 Table — (DOCX) [file pone.0120326.s009.docx]

**Table 8. Leading edge of GSEA comparing *MLL*-rearranged patients versus wild-type *MLL* patients using 57 MLL-AF4 target gene probe sets (Figure 4B)**

| Probe set | HGNC Gene Symbol |
| --- | --- |
| 204304_s_at | PROM1 |
| 232544_at | IGFBP7 |
| 212080_at | MLL |
| 235753_at | HOXA7 |
| 206847_s_at | HOXA7 |
| 236443_at | PAX5 |
| 1553145_at | FLJ39653 |
| 233931_at | ZFR |
| 203753_at | TCF4 |
| 1564776_at | 1564776_at |
| 203216_s_at | MYO6 |
| 243001_at | C18orf22 |
| 1568589_at | REEP3 |
| 207143_at | CDK6 |
| 235479_at | CPEB2 |
