# Supplementary material for: Identification of Genes Transcriptionally Responsive to the Loss of MLL Fusions in MLL-Rearranged Acute Lymphoblastic Leukemia
Source: PLoS One. 2015 Mar 20;10(3):e0120326. doi: 10.1371/journal.pone.0120326 (PMC4368425; doi:10.1371/journal.pone.0120326)
Supplement: S9 Table — (DOCX) [file pone.0120326.s010.docx]

**Table 9. Differentially expressed genes in response to the repression of MLL-AF4 and MLL-ENL as compared to the si*AGF1* control (n=80) or the pulse control (no siRNAs) (n=58) (Figure 5B)**

**80 probe sets: 58 probe sets:**

***AF4-MLL* KD versus *AGF1* control *AF4-MLL* KD versus pulse control**

| Probe set | HGNC Gene Symbol | logFC | P.Value |  | Probe set | HGNC Gene Symbol | logFC | P.Value |
| --- | --- | --- | --- | --- | --- | --- | --- | --- |
| 222631_at | PI4K2B | -0.58 | 3.21E-07 |  | 212636_at | QKI | -0.89 | 1.14E-06 |
| 212636_at | QKI | -0.87 | 5.29E-07 |  | 222631_at | PI4K2B | -0.64 | 2.31E-06 |
| 209312_x_at | NA | 0.45 | 1.42E-06 |  | 226297_at | HIPK3 | -0.72 | 3.91E-06 |
| 226297_at | HIPK3 | -0.72 | 1.57E-06 |  | 225935_at | CUX1 | -0.36 | 4.82E-06 |
| 228486_at | SLC44A1 | -0.45 | 2.98E-06 |  | 204517_at | PPIC | 0.41 | 1.35E-05 |
| 204670_x_at | NA | 0.40 | 3.23E-06 |  | 222870_s_at | B3GNT2 | -0.56 | 1.89E-05 |
| 228008_at | NA | -0.33 | 5.30E-06 |  | 219326_s_at | B3GNT2 | -0.55 | 2.86E-05 |
| 208306_x_at | NA | 0.39 | 6.16E-06 |  | 217910_x_at | MLX | -0.30 | 3.44E-05 |
| 219326_s_at | B3GNT2 | -0.45 | 6.97E-06 |  | 212750_at | PPP1R16B | 0.40 | 3.98E-05 |
| 213708_s_at | MLX | -0.36 | 8.64E-06 |  | 41577_at | PPP1R16B | 0.42 | 4.59E-05 |
| 215193_x_at | NA | 0.42 | 1.06E-05 |  | 213708_s_at | MLX | -0.35 | 5.09E-05 |
| 222870_s_at | B3GNT2 | -0.60 | 1.14E-05 |  | 201858_s_at | SRGN | 0.54 | 5.15E-05 |
| 223343_at | MS4A7 | 0.42 | 1.28E-05 |  | 223017_at | TXNDC12 | -0.46 | 7.93E-05 |
| 222958_s_at | DEPDC1 | -0.56 | 1.93E-05 |  | 238041_at | TCF12 | -0.37 | 8.67E-05 |
| 41577_at | PPP1R16B | 0.39 | 2.12E-05 |  | 205632_s_at | PIP5K1B | 0.29 | 1.36E-04 |
| 208724_s_at | RAB1A | -0.35 | 2.60E-05 |  | 201859_at | SRGN | 0.42 | 1.45E-04 |
| 226689_at | CISD2 | -0.42 | 3.66E-05 |  | 222958_s_at | DEPDC1 | -0.59 | 1.53E-04 |
| 217910_x_at | MLX | -0.27 | 5.38E-05 |  | 236513_at | NA | -0.31 | 1.55E-04 |
| 233727_at | NA | 0.35 | 5.85E-05 |  | 204518_s_at | PPIC | 0.32 | 1.60E-04 |
| 236513_at | NA | -0.30 | 5.87E-05 |  | 208724_s_at | RAB1A | -0.29 | 1.62E-04 |
| 204249_s_at | LMO2 | 0.45 | 6.07E-05 |  | 228486_at | SLC44A1 | -0.51 | 1.95E-04 |
| 227699_at | C14orf149 | -0.31 | 6.07E-05 |  | 229844_at | FOXP1 | -0.27 | 3.02E-04 |
| 211990_at | HLA-DPA1 | 0.29 | 7.34E-05 |  | 226793_at | LINC00294 | -0.25 | 3.30E-04 |
| 225935_at | CUX1 | -0.29 | 7.47E-05 |  | 209789_at | CORO2B | -0.37 | 3.45E-04 |
| 211924_s_at | PLAUR | 0.30 | 7.52E-05 |  | 214743_at | CUX1 | -0.31 | 3.59E-04 |
| 212750_at | PPP1R16B | 0.35 | 1.00E-04 |  | 233727_at | NA | 0.30 | 3.74E-04 |
| 223047_at | CMTM6 | -0.35 | 1.13E-04 |  | 232278_s_at | DEPDC1 | -0.54 | 4.08E-04 |
| 214866_at | PLAUR | 0.23 | 1.16E-04 |  | 209711_at | SLC35D1 | -0.26 | 4.51E-04 |
| 217947_at | CMTM6 | -0.36 | 1.38E-04 |  | 223171_at | DYM | -0.57 | 4.52E-04 |
| 229307_at | ANKRD28 | 0.32 | 1.64E-04 |  | 1552726_at | ADAMTS17 | 0.27 | 4.53E-04 |
| 226793_at | LINC00294 | -0.27 | 1.65E-04 |  | 235545_at | DEPDC1 | -0.58 | 4.70E-04 |
| 223171_at | DYM | -0.45 | 1.65E-04 |  | 203725_at | GADD45A | -0.29 | 4.83E-04 |
| 235534_at | NA | -0.24 | 1.69E-04 |  | 204891_s_at | LCK | -0.30 | 4.89E-04 |
| 223017_at | TXNDC12 | -0.43 | 1.72E-04 |  | 203320_at | SH2B3 | 0.28 | 5.01E-04 |
| 211654_x_at | HLA-DQB1 | 0.32 | 1.82E-04 |  | 226689_at | CISD2 | -0.31 | 5.19E-04 |
| 238759_at | CCDC88A | 0.26 | 1.94E-04 |  | 212262_at | QKI | -0.23 | 5.40E-04 |
| 217974_at | TM7SF3 | -0.31 | 2.22E-04 |  | 225262_at | FOSL2 | 0.23 | 5.47E-04 |
| 235545_at | DEPDC1 | -0.54 | 2.24E-04 |  | 204249_s_at | LMO2 | 0.40 | 5.50E-04 |
| 211656_x_at | HLA-DQB1 | 0.36 | 2.31E-04 |  | 228094_at | AMICA1 | 0.27 | 5.59E-04 |
| 1553601_a_at | TMIE | 0.25 | 2.35E-04 |  | 1554343_a_at | STAP1 | 0.29 | 5.66E-04 |
| 208894_at | HLA-DRA | 0.38 | 2.37E-04 |  | 222942_s_at | NA | 0.24 | 5.85E-04 |
| 212262_at | QKI | -0.27 | 2.53E-04 |  | 221786_at | C6orf120 | 0.24 | 5.97E-04 |
| 232278_s_at | DEPDC1 | -0.48 | 2.75E-04 |  | 210845_s_at | PLAUR | 0.27 | 6.66E-04 |
| 209789_at | CORO2B | -0.43 | 2.79E-04 |  | 214866_at | PLAUR | 0.22 | 6.75E-04 |
| 211991_s_at | HLA-DPA1 | 0.29 | 2.87E-04 |  | 221595_at | C7orf64 | -0.23 | 6.77E-04 |
| 239400_at | NA | 0.27 | 2.94E-04 |  | 212080_at | MLL | -0.53 | 7.28E-04 |
| 234915_s_at | DENR | -0.26 | 2.95E-04 |  | 209712_at | SLC35D1 | -0.29 | 7.34E-04 |
| 210982_s_at | HLA-DRA | 0.40 | 3.00E-04 |  | 218446_s_at | FAM18B1 | 0.30 | 7.41E-04 |
| 242520_s_at | C1orf228 | 0.41 | 3.33E-04 |  | 228008_at | NA | -0.34 | 7.59E-04 |
| 222248_s_at | SIRT4 | -0.19 | 3.48E-04 |  | 204174_at | ALOX5AP | 0.33 | 8.10E-04 |
| 226981_at | MLL | -0.61 | 3.73E-04 |  | 224967_at | UGCG | 0.39 | 8.13E-04 |
| 213975_s_at | LYZ | 0.28 | 3.94E-04 |  | 218017_s_at | HGSNAT | -0.23 | 8.66E-04 |
| 224391_s_at | SIAE | 0.32 | 4.07E-04 |  | 210752_s_at | MLX | -0.20 | 8.81E-04 |
| 203416_at | CD53 | 0.26 | 4.09E-04 |  | 210140_at | CST7 | 0.40 | 9.15E-04 |
| 222942_s_at | NA | 0.22 | 4.11E-04 |  | 239400_at | NA | 0.26 | 9.28E-04 |
| 223060_at | C14orf119 | -0.27 | 4.25E-04 |  | 227069_at | CUX1 | -0.17 | 9.41E-04 |
| 228094_at | AMICA1 | 0.26 | 4.58E-04 |  | 238873_at | NA | -0.18 | 9.70E-04 |
| 218865_at | MOSC1 | -0.25 | 4.66E-04 |  | 211924_s_at | PLAUR | 0.25 | 9.79E-04 |
| 228135_at | C1orf52 | -0.51 | 5.01E-04 |  |  |  |  |  |
| 207791_s_at | RAB1A | -0.32 | 5.14E-04 |  |  |  |  |  |
| 226946_at | NADKD1 | 0.27 | 5.36E-04 |  |  |  |  |  |
| 228783_at | BVES | -0.30 | 5.39E-04 |  |  |  |  |  |
| 202804_at | ABCC1 | 0.36 | 5.55E-04 |  |  |  |  |  |
| 227069_at | CUX1 | -0.20 | 5.79E-04 |  |  |  |  |  |
| 212080_at | MLL | -0.39 | 6.24E-04 |  |  |  |  |  |
| 238041_at | TCF12 | -0.31 | 6.33E-04 |  |  |  |  |  |
| 1554712_a_at | GLYATL2 | 0.25 | 6.39E-04 |  |  |  |  |  |
| 218668_s_at | RAP2C | -0.29 | 6.66E-04 |  |  |  |  |  |
| 224967_at | UGCG | 0.32 | 6.79E-04 |  |  |  |  |  |
| 227819_at | LGR6 | -0.31 | 7.29E-04 |  |  |  |  |  |
| 201778_s_at | KIAA0494 | -0.34 | 7.33E-04 |  |  |  |  |  |
| 214743_at | CUX1 | -0.29 | 7.54E-04 |  |  |  |  |  |
| 224691_at | UHMK1 | -0.28 | 7.63E-04 |  |  |  |  |  |
| 228345_at | CHIC1 | -0.34 | 8.10E-04 |  |  |  |  |  |
| 227809_at | ZC3H6 | 0.22 | 8.80E-04 |  |  |  |  |  |
| 204635_at | RPS6KA5 | -0.18 | 8.92E-04 |  |  |  |  |  |
| 225784_s_at | ZC4H2 | -0.20 | 9.03E-04 |  |  |  |  |  |
| 212771_at | FAM171A1 | -0.19 | 9.42E-04 |  |  |  |  |  |
| 224818_at | SORT1 | -0.29 | 9.63E-04 |  |  |  |  |  |
