# Supplementary material for: Identification of Genes Transcriptionally Responsive to the Loss of MLL Fusions in MLL-Rearranged Acute Lymphoblastic Leukemia
Source: PLoS One. 2015 Mar 20;10(3):e0120326. doi: 10.1371/journal.pone.0120326 (PMC4368425; doi:10.1371/journal.pone.0120326)
Supplement: S11 Table — (DOCX) [file pone.0120326.s012.docx]

Table 11. Down-regulated genes in *MLL-AF4* signature (Figure 6B, lower panel)

| Probe set | HGNC Gene Symbol |
| --- | --- |
| 1557192_at | NA |
| 201776_s_at | KIAA0494 |
| 201778_s_at | KIAA0494 |
| 203725_at | GADD45A |
| 204045_at | TCEAL1 |
| 205771_s_at | AKAP7 |
| 206498_at | OCA2 |
| 207791_s_at | RAB1A |
| 208724_s_at | RAB1A |
| 209711_at | SLC35D1 |
| 209789_at | CORO2B |
| 210752_s_at | MLX |
| 212080_at | MLL |
| 212262_at | QKI |
| 212636_at | QKI |
| 213227_at | PGRMC2 |
| 213708_s_at | MLX |
| 214743_at | CUX1 |
| 215001_s_at | GLUL |
| 217910_x_at | MLX |
| 217947_at | CMTM6 |
| 217974_at | TM7SF3 |
| 217982_s_at | MORF4L1 |
| 218865_at | MOSC1 |
| 219326_s_at | B3GNT2 |
| 222631_at | PI4K2B |
| 222870_s_at | B3GNT2 |
| 222958_s_at | DEPDC1 |
| 223017_at | TXNDC12 |
| 223047_at | CMTM6 |
| 223060_at | C14orf119 |
| 223171_at | DYM |
| 224818_at | SORT1 |
| 225784_s_at | ZC4H2 |
| 225935_at | NA |
| 226297_at | NA |
| 226478_at | TM7SF3 |
| 226689_at | CISD2 |
| 226793_at | LOC283267 |
| 226868_at | GLT8D3 |
| 226980_at | DEPDC1B |
| 226981_at | MLL |
| 227069_at | NA |
| 227699_at | C14orf149 |
| 228008_at | NA |
| 228135_at | C1orf52 |
| 228345_at | CHIC1 |
| 228486_at | SLC44A1 |
| 228540_at | QKI |
| 228783_at | BVES |
| 229844_at | NA |
| 231896_s_at | DENR |
| 232278_s_at | DEPDC1 |
| 235545_at | DEPDC1 |
| 236513_at | NA |
| 238041_at | NA |
| 238756_at | GAS2L3 |
| 238873_at | NA |
