# Supplementary material for: Identification of Genes Transcriptionally Responsive to the Loss of MLL Fusions in MLL-Rearranged Acute Lymphoblastic Leukemia
Source: PLoS One. 2015 Mar 20;10(3):e0120326. doi: 10.1371/journal.pone.0120326 (PMC4368425; doi:10.1371/journal.pone.0120326)
Supplement: S12 Table — (DOCX) [file pone.0120326.s013.docx]

**Table 12. Leading edge of GSEA comparing AF4-MLL positive patients versus AF4-MLL negative t(4;11) patients using 58 AF4-MLL target gene probe sets (Figure 6B, upper panel)**

| Probe set | HGNC Gene Symbol |
| --- | --- |
| 226981_at | MLL |
| 212080_at | MLL |
| 206498_at | OCA2 |
| 217974_at | TM7SF3 |
| 222958_s_at | DEPDC1 |
| 228345_at | CHIC1 |
| 226868_at | GXYLT1 |
| 236513_at | PRELID2 |
| 238041_at | TCF12 |
| 222631_at | PI4K2B |
| 226689_at | CISD2 |
| 210752_s_at | MLX |
| 226980_at | DEPDC1B |
| 227069_at | CUX1 |
| 223060_at | C14orf119 |
