# Supplementary material for: Identification of Genes Transcriptionally Responsive to the Loss of MLL Fusions in MLL-Rearranged Acute Lymphoblastic Leukemia
Source: PLoS One. 2015 Mar 20;10(3):e0120326. doi: 10.1371/journal.pone.0120326 (PMC4368425; doi:10.1371/journal.pone.0120326)
Supplement: S13 Table — (DOCX) [file pone.0120326.s014.docx]

**Table 13. Leading edge of GSEA comparing MLL fusion knockdown versus control samples using dataset from Stumpel *et al*  (Figure 7)**

| Probe set | HGNC Gene Symbol |
| --- | --- |
| 1553264_a_at | SYN1 |
| 1554112_a_at | ULK2 |
| 1554980_a_at | ATF3 |
| 1570071_at | MYO15A |
| 202388_at | RGS2 |
| 203898_at | CRCP |
| 204739_at | CENPC1 |
| 204924_at | TLR2 |
| 206299_at | FAM155B |
| 206906_at | ICAM5 |
| 206908_s_at | CLDN11 |
| 207373_at | HOXD10 |
| 207692_s_at | ACAN |
| 208605_s_at | NTRK1 |
| 208942_s_at | SEC62 |
| 209119_x_at | NR2F2 |
| 210051_at | RAPGEF3 |
| 210673_x_at | NKX2.1 |
| 211170_s_at | PDE10A |
| 216971_s_at | PLEC |
| 219527_at | MOSC2 |
| 220487_at | SNTG2 |
| 221015_s_at | CDADC1 |
| 221608_at | WNT6 |
| 222200_s_at | BSDC1 |
| 226178_at | SOCS4 |
| 227062_at | NEAT1 |
| 233052_at | DNAH8 |
| 233287_at | SLC6A17 |
| 234721_s_at | CYP26B1 |
